# Construction of the optimization prognostic model based on differentially expressed immune genes of lung adenocarcinoma

**DOI:** 10.1186/s12885-021-07911-8

**Published:** 2021-03-01

**Authors:** Yang Zhai, Bin Zhao, Yuzhen Wang, Lina Li, Jingjin Li, Xu Li, Linhan Chang, Qian Chen, Zijun Liao

**Affiliations:** 1 Department of Oncology, Tumor Hospital of Shaanxi Province, Xi’an, 710061 People’s Republic of China; 2 Bioinspired Engineering and Biomechanics Center (BEBC), Xi’an Jiaotong University, Xi’an, 710049 PR China; 3 Department of Epidemiology, Shaanxi Provincial Tumor Hospital, Xi’an, 710061 China; 4 The Key Laboratory of Biomedical Information Engineering of Ministry of Education, School of Life Science and Technology, Xi’an Jiaotong University, Xi’an, 710049 China; 5 Department of Vasculocardiology, First Affiliated Hospital, Xi’an Jiaotong University Medical College, Xi’an, 710061 PR China; 6 Xi’an Medical University, Xi’an, 710061 PR China; 7 Department of Reproduction, First Affiliated Hospital, Xi’an Jiaotong University Medical College, Xi’an, Shaanxi 710061 PR China

**Keywords:** Immunogenomics, TCGA, Lung adenocarcinoma, Prognosis, Optimization models

## Abstract

**Background:**

Lung adenocarcinoma (LUAD) is the most common pathology subtype of lung cancer. In recent years, immunotherapy, targeted therapy and chemotherapeutics conferred a certain curative effects. However, the effect and prognosis of LUAD patients are different, and the efficacy of existing LUAD risk prediction models is unsatisfactory.

**Methods:**

The Cancer Genome Atlas (TCGA) LUAD dataset was downloaded. The differentially expressed immune genes (DEIGs) were analyzed with edgeR and DESeq2. The prognostic DEIGs were identified by COX regression. Protein-protein interaction (PPI) network was inferred by STRING using prognostic DEIGs with *p* value< 0.05. The prognostic model based on DEIGs was established using Lasso regression. Immunohistochemistry was used to assess the expression of FERMT2, FKBP3, SMAD9, GATA2, and ITIH4 in 30 cases of LUAD tissues.

**Results:**

In total,1654 DEIGs were identified, of which 436 genes were prognostic. Gene functional enrichment analysis indicated that the DEIGs were involved in inflammatory pathways. We constructed 4 models using DEIGs. Finally, model 4, which was constructed using the 436 DEIGs performed the best in prognostic predictions, the receiver operating characteristic curve (ROC) was 0.824 for 3 years, 0.838 for 5 years, 0.834 for 10 years. High levels of FERMT2, FKBP3 and low levels of SMAD9, GATA2, ITIH4 expression are related to the poor overall survival in LUAD (*p* < 0.05). The prognostic model based on DEIGs reflected infiltration by immune cells.

**Conclusions:**

In our study, we built an optimal prognostic signature for LUAD using DEIGs and verified the expression of selected genes in LUAD. Our result suggests immune signature can be harnessed to obtain prognostic insights.

**Supplementary Information:**

The online version contains supplementary material available at 10.1186/s12885-021-07911-8.

## Introduction

Lung cancer is one of the most common diseases with the highest morbidity and mortality, in which the lung adenocarcinoma accounts for 40% of all cases. In recent years, the morbidity and mortality of lung adenocarcinoma have gradually increased [1]. Chemotherapy, radiotherapy and targeted therapy are the most common therapeutic methods for advanced lung adenocarcinoma. Although multiple therapeutics have been used in LUAD, the overall effective rate is unsatisfactory.

Increasing evidence suggested that tumor microenvironment (TME) which is composed of tumor cells, immune cells, stromal cells, inflammatory mediators and extracellular matrix [2], taking part in the tumor progression and drug resistance [3, 4]. Among them, immune cells and inflammatory mediators have been proved to be valuable for the prognostic of LUAD [5]. Much attention has been paid on the immune microenvironment of LUAD.

Current studies showed that immunology and immunogenomics were closely tied to the development of LUAD [6, 7]. Immunotherapy is expected to replace the traditional treatment based on a number of clinical studies. In recent years, the emergence of immune checkpoint inhibitors has enabled a dramatic progress in cancer treatment [8, 9]. How to select the patients who really benefit from immunotherapy has become an urgent problem to be solved. It is important to identify biomarkers that can predict disease prognosis and identify the patients who have the greatest curative effect. S-PD-L1 and T-PD-1 were verified as the independent prognostic factors for non small-cell lung cancer (NSCLC) patients by Paulsen [10]. Their combination added significant prognostic impact within each pathologic stage. Several studies suggested that tumor mutational burden (TMB) [11, 12], mismatch repair (MMR) [13, 14] are new biomarkers for prediction of response to PD-L1 treatment. However, cause of the heterogeneity, accurate theranostic biomarkers are still lacking. The exploration of biomarkers in the immune microenvironment remains largely unknown. In this study, we combined multiple datasets from TCGA LUAD to develop and validate a prognosis prediction model for LUAD. Meanwhile, an optimal prognostic model with the identified DEIGs via lasso regression was established by us. Our aim is to give a more in-depth view of the prognostic potential of DEIGs in clinical and provides a foundation for future, in-depth immune-related work of LUAD.

## Materials and methods

All methods were carried out in accordance with relevant guidelines and regulations.

### Data preprocessing

TCGA LUAD dataset legacy-archive (hg19) was downloaded from NCI’s Genomic Data Commons (GDC) (https://portal.gdc.cancer.gov) using R package ‘TCGA biolinks’ [15], and only “Primary solid Tumor” and “Solid Tissue Normal” samples were included. Furthermore, the immune-related genes were derived from InnateDB (https://www.innatedb.com) [16]. While the estimated infiltration abundance of immune cells of LUAD samples were obtained by TIMER (https://cistrome.shinyapps.io/timer/) [17].TIMER is a resource providing pre-calculated levels of six tumor-infiltrating immune subsets for 10,897 tumors from 32 cancer types.

### Identification of prognostic DEIGs

Differentially expressed RNAs were detected using DESeq2 [18] and edgeR [19]. RNAs with ‘|log2 (fold change) | > 1’, ‘*p* value < 0.05’ and ‘fdr < 0.3’ in both methods were considered to be differentially expressed. COX regression was employed to identify prognostic DEIGs.

### Annotation of prognostic DEIGs

The R package ‘ClusterProfiler’ [20] was employed for pathway enrichment analysis with DEIGs. Functional enrichment analyses, via the Gene Ontology (GO) and Kyoto Encyclopedia of Genes and Genomes (KEGG) pathways [21], were conducted to explore potential molecular mechanisms of the differentially expressed prognostic DEIGs.

### PPI network construction and hub-genes identification

PPI network was inferred by STRING using the prognostic differentially expressed immune genes with *p* value< 0.05 in cox test [22]. Hub genes were identified by cytoscape.

### Modeling via lasso regression

We used glmnet package to fit regularized Cox models. The function cv.glmnet was used to compute K-fold cross-validation (CV) for the Cox model with parameters ‘ family=“cox”, nfolds=10’. The optimal λ value and a cross validated error plot were shown as below. The left vertical line indicated where the CV-error curve hits its minimum. And the right vertical line showed the most regularized model with CV-error within 1 standard deviation of the minimum. We then extracted the lambda.min for model construction.

The whole TCGA dataset was divided into 70% of training samples and 30% of test samples. The prediction model was built on the most frequent gene set with effective coefficients in the lasso regression using R package ‘glmnet’ [23] for 1000 iterations on the training dataset. The risk score was defined as the sum of the normalized expression of genes multiplied by their coefficients in the gene set. ROC was used to evaluate the cutoff of risk scores as a predicting factor for the survival of LUAD patients at 5 years prior to death. After dividing the patient into two groups according to the risk score, ‘Survminer’ was employed for survival analysis for both training and testing data. The pearson correlation coefficients of risk score and immune cells/immune cells markers were calculated by the R package ‘ggpubr’.

### Immunohistochemistry

This study recruited 30 patients of LUAD getting surgery at Tumor Hospital of Shaanxi province between January 2014 and December 2015 whom had no prior chemotherapy or radiotherapy. Antibodies included a rabbit polyclonal anti-FERMT2 antibody at a dilution of 1:50, anti- FKBP3 antibody at a dilution of 1:50, (all from Proteintech Group, China), anti-SMAD9 antibody at a dilution of 1:100, anti-GATA2 antibody at a dilution of 1:100, anti-ITIH4 antibody at a dilution of 1:50 (all from Beijing Biosynthesis Biotechnology, China). PBS was used to displace the primary antibody as the negative control. The histological diagnosis was performed by 3 independent, experienced pathologists for all the cases. The Immunohistochemistry (IHC) was performed according to our previous study [24]. Five micrometer-thick sections were cut from the human lung adenocarcinoma tissue and fixed in 10% buffered formalin overnight and paraffin-embedded. The slides were deparaffinized and rehydrated in graded alcohols, followed by antigen retrieval in a microwave oven. Slides were blocked with 10% normal goat serum for 20 min at 37 °C to reduce nonspecific binding. The slides were incubated overnight at 4 °C [25]. After being washed, Horseradish peroxidase (HRP) conjugated goat anti-rabbit IgG was used as secondary antibody, and then visualized with 3,3′-diaminobenzidine (DAB) solution. Finally, hematoxylin was used to counterstain the section. The percentage of positive cells was classified into 5 score ranges: < 10% (0),10 to 25% (1), 25 to 50% (2), 50 to 75% (3), and > 75% (4). The intensity of staining was divided into 4 groups: no staining (0), light brown (1), brown (2), and dark brown (3). The staining positivity was determined using immunoreactivity score (IRS) which is the product of intensity score and quantity score. An overall score of > 6 as strong positive, > 3 as weak positive, and ≤ 3 was defined as negative.

## Results

### Identification of prognostic DEIGs

The immune-related genes were downloaded from InnateDB. The differentially expressed gene analysis was performed by edgeR and DESeq2, and only DEIGs detected by both methods were included. Four hundred thirty-six genes were identified with *p* value <= 0.05 in cox tests by the R package ‘survival’ (Table S1).

### PPI network and hub genes

To gain insights into the core pathways exerted by those DEIGs, we constructed PPI network and identified core modules within the network. PPI analysis demonstrated that FANCI, MAD2L1, ECT2, PLK4, PCNA, BUB1B, RACGAP1, PRC1, CDK1, TACC3, MCM7, EXO1, TPX2, BUB1, ANLN, ESPL1, KPNA2, AURKB, FEN1, NUSAP1, CCNB2, HMMR, CKAP2, INCENP, MKI67, BIRC5, HELLS, ZWILCH, TOP2A, ERCC6L and INCENP were the hub genes (Figure S1).

### Characteristics of prognostic DEIGs

**Fig. 1:**
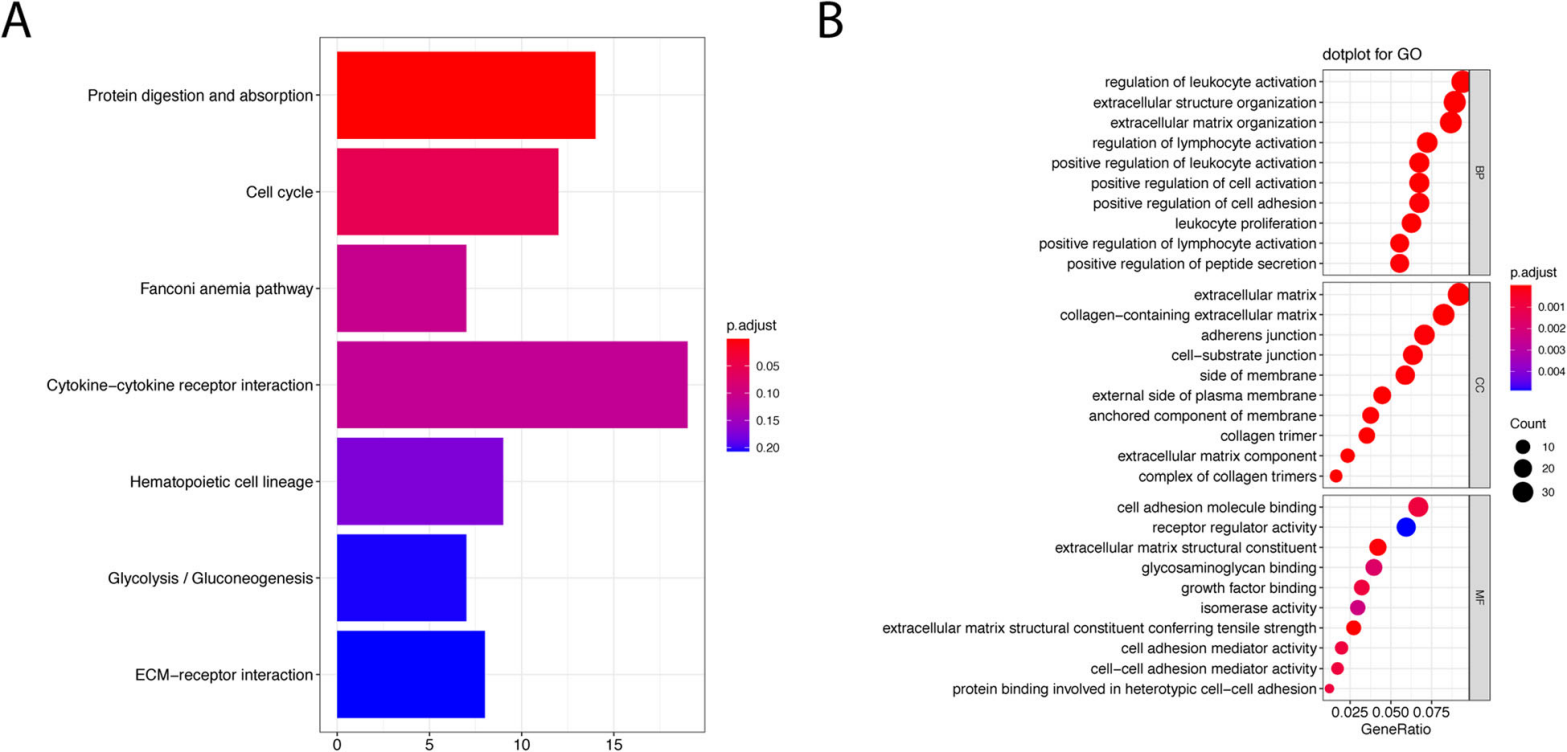
Gene functional enrichment of differentially expressed immune-related genes (**a**Top10 enriched KEGG gene sets; **b** GO analyses of the prognostic DEGs in the categories of biological processes (BP), cellular components (CC), and molecular functions (MF))

As expected, the inflammatory pathways were indicated as the most frequently implicated by gene functional enrichment analysis. Regulation of leukocyte activation, extracellular matrix and cell adhesion molecule binding were the most frequent GO terms (Fig. 1a). The cytokine−cytokine receptor interaction was the top term enriched by differentially expressed prognostic DEIGs (Fig. 1b). We also found that the missense is the most common type of mutations by examining genetic alterations of these genes (Figure S2).

### Prognostic modeling, identification of an optimal prognostic signature using immune related genes

**Fig. 2:**
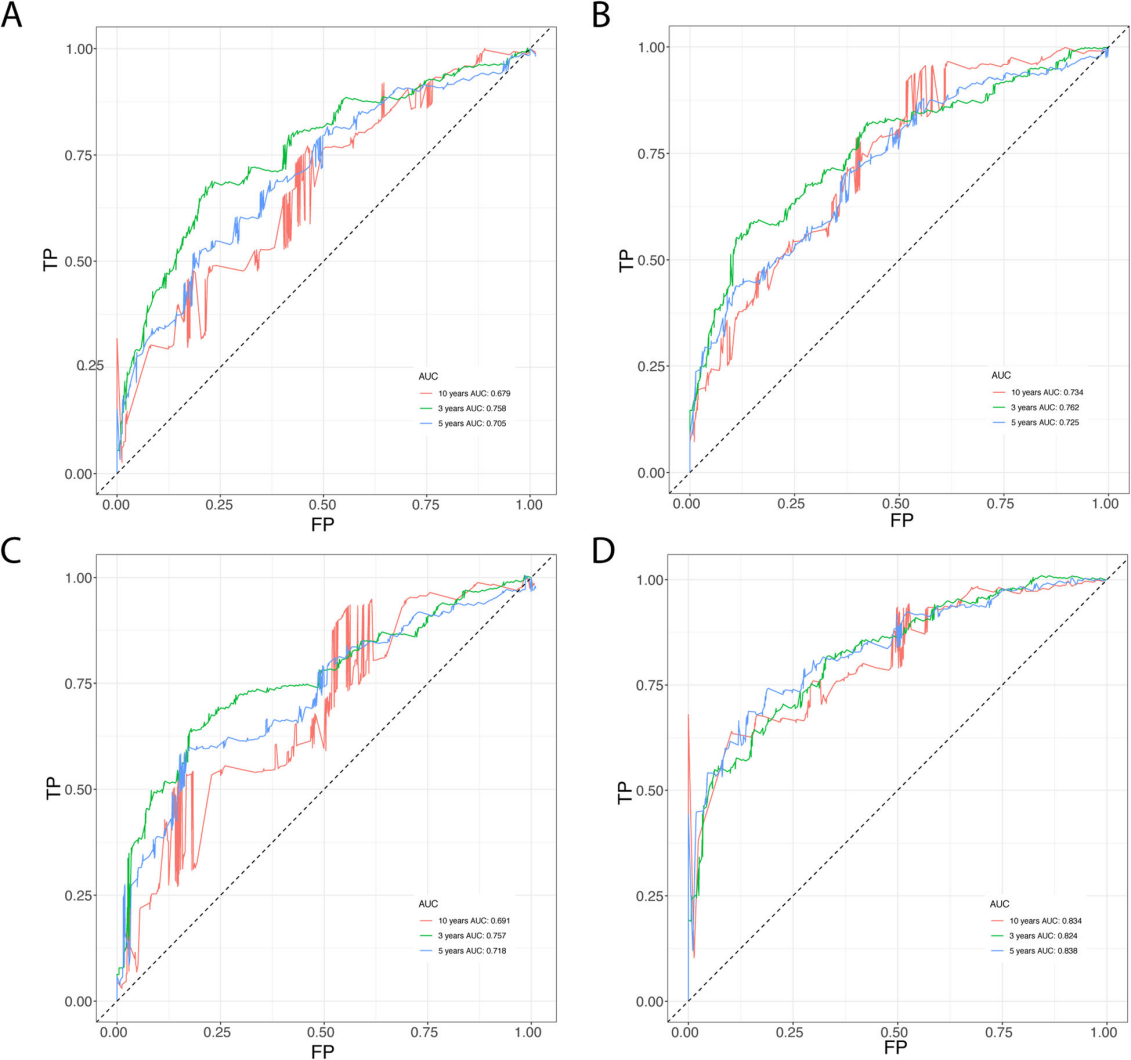
ROC curve validation of prognostic value of the prognostic index of each model (**a** Modle-1, Input gene list: top100 sorted immune-related genes by *p* value; **b** Modle-2, Input gene list: top159 sorted immune-related genes by *p* value; **c** Modle-3, Input gene list: top200 sorted immune-related genes by *p* value; **d** Modle-4, Input gene list: top436sorted immune-related genes by *p* value)

**Fig. 3:**
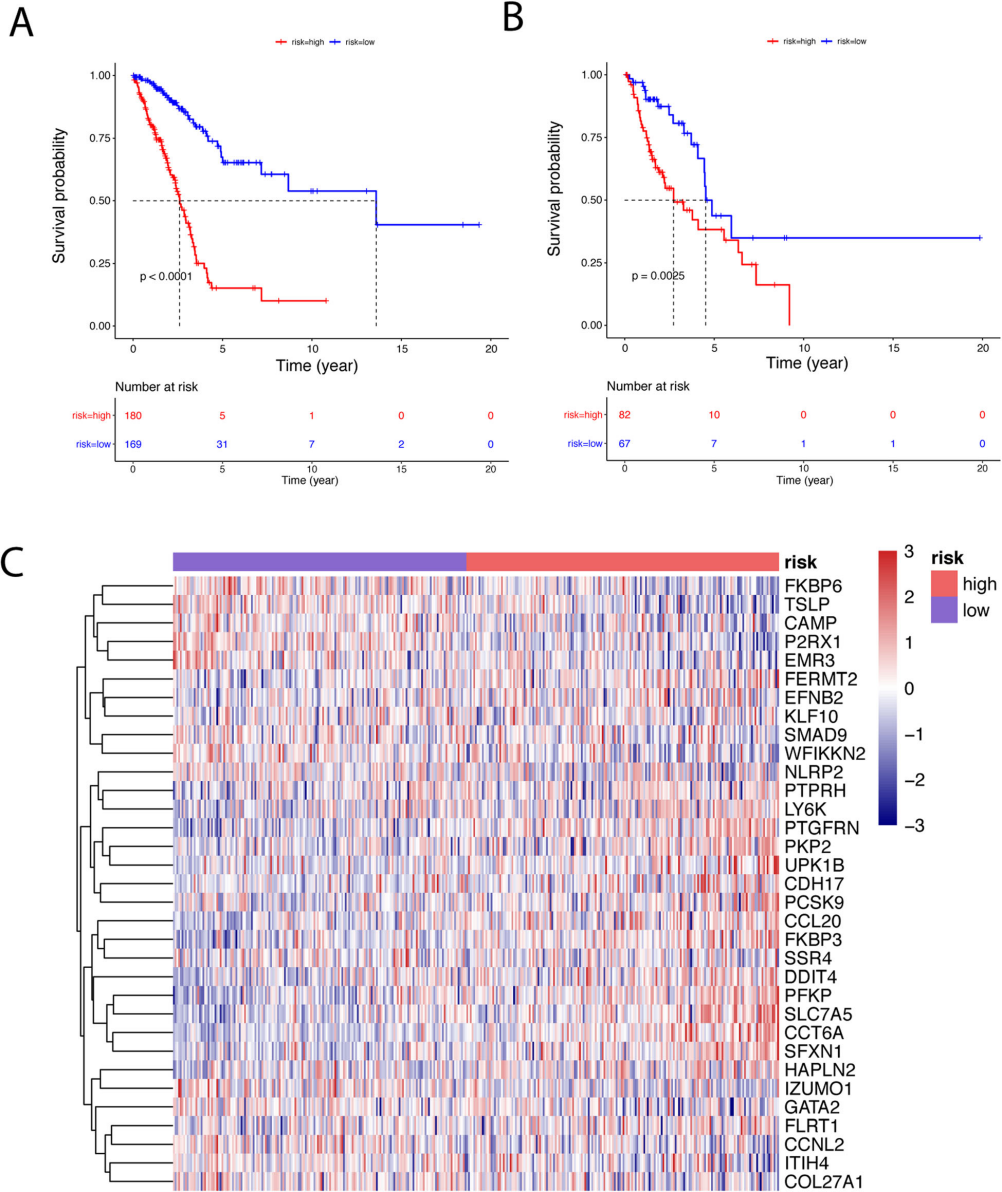
Identification of an immune signature predicting prognosis risk of patients in LUAD using model 4 (**a** survival analysis of the training dataset; **b** survival analysis in the testing data; **c** The heatmaps distinct gene expression profiles of the cases belonging to the high and low risk score groups)

**Fig. 4:**
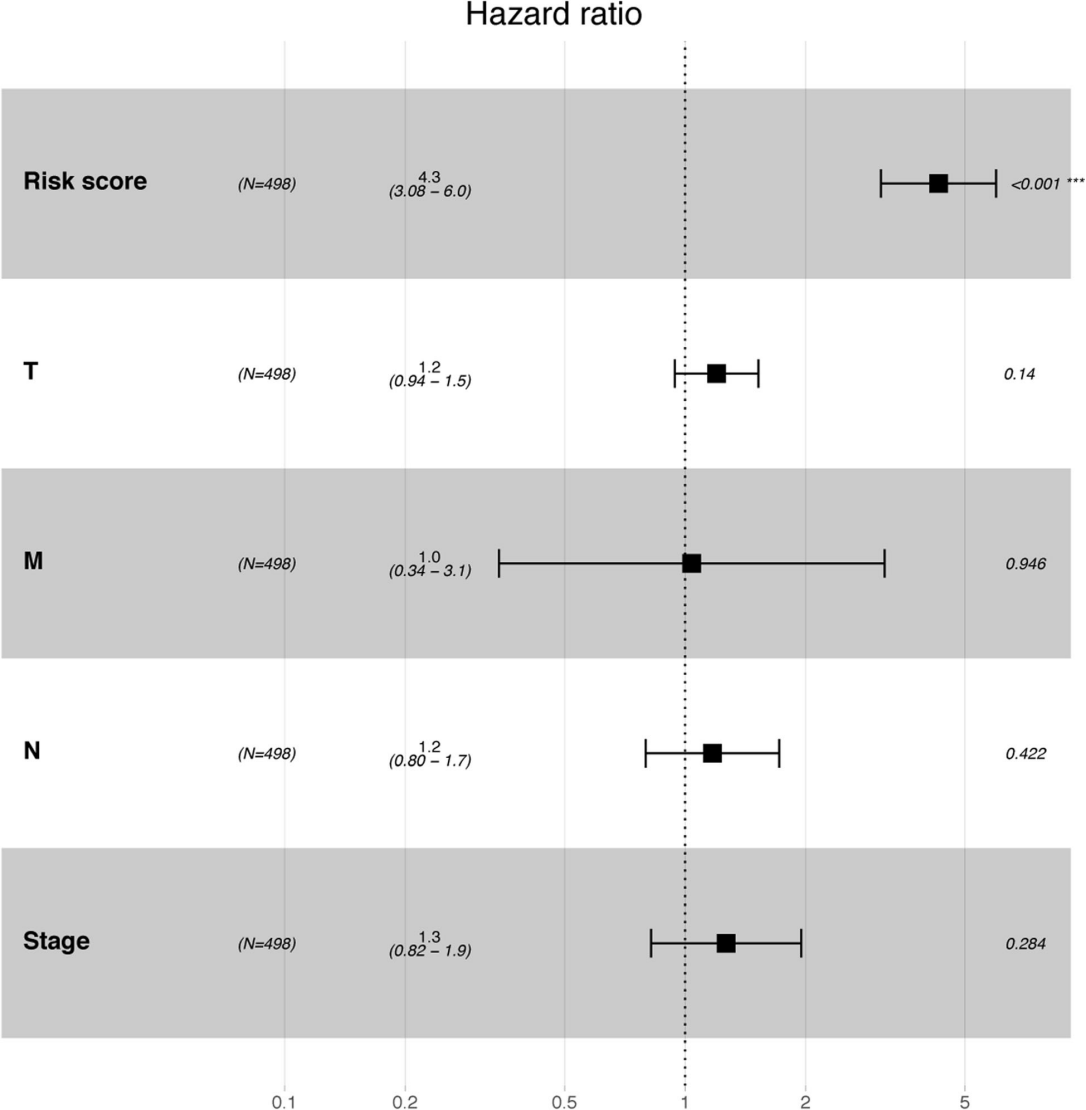
Multivariate cox regression analysis

The prediction model was built on the most frequent gene set with effective coefficients in the lasso regression. Model 1, Model 2, Model 3 and Model 4 were respectively constructed using the top 100, 159, 200 and 436 DEIGs. We found that model 4 which was correlated with tumor burden, tumor stage and metastasis, performed best in prognostic predictions. The optimized model consists of the following genes: CAMP, CCT6A, CDH17, EFNB2, FKBP3, GATA2, ITIH4, SMAD9, P2RX1, PFKP, PKP2, PTGFRN, PTPRH, CCL20, SSR4, KLF10, UPK1B, SLC7A5, FKBP6, FERMT2, FLRT1, DDIT4, LY6K, NLRP2, HAPLN2, CCNL2, EMR3, COL27A1, TSLP, SFXN1, WFIKKN2, PCSK9, IZUMO1. The list of coefficients for those genes are shown in Supplementary Information (Table S2, Figure S3). The ROC curve was 0.824 for 3 years, 0.838 for 5 years, 0.834 for 10 years, indicating the prognostic model based on DEIGs has definite potential in survival monitoring (Figs. 2 and 3, S4). Univariate Cox regression analysis suggested that the prognostic signature, age, tumor stage, pathologic stage and metastasis status are all associated with prognosis (Table 1). The prognostic model based on DEIGs was identified as an independent predictor by using multivariate cox regression analysis after the adjustment of other parameters (Fig. 4).

**Table 1:** Univariate cox regression analysis

	HR	95%CI	***P*** value
Riskscore	4.6	(3.5–6.1)	4.5e-28
TMB	0.97	(0.9–1)	0.46
T	1.5	(1.3–1.8)	7.8e-06
M	2.2	(1.3–3.7)	0.0047
N	1.7	(1.4–2)	1.9e-09
Age	1	(0.99–1)	0.48
Stage	1.7	(1.5–1.9)	4.9e-13
Smoke	0.92	(0.67–1.3)	0.59
Gender	1.1	(0.79–1.4)	0.68

### Correlation between prognostic signature and immune infiltration

**Fig. 5:**
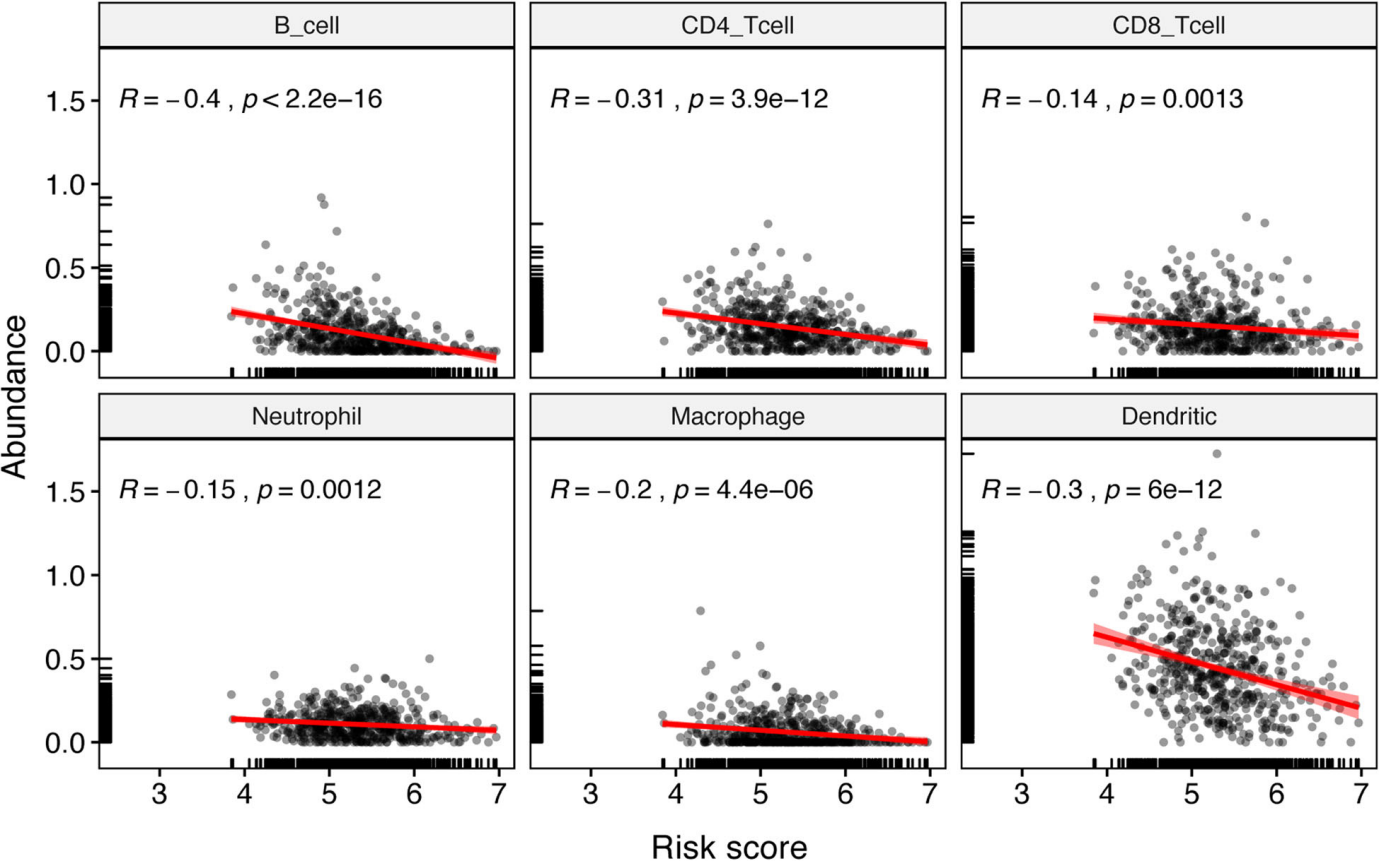
Relationships between the risk score and estimated infiltration abundances of immune cells

We analyzed the relationship between model predicted risk score and immune cell infiltration to see if the DEIGs accurately reflected the status of tumor immune microenvironment. The risk score of our model is inversely related to the abundances of infiltrated immune cells as well as classical markers for immune cells, including CD8+ T cell, CD4+ T cell, B cell and dendritic cell (Fig. 5, S5, S6, S7 and S8).

### The relationship between the expression of FERMT2, FKBP3, SMAD9, GATA2, IHIH4 and the overall survival of LUAD

**Fig. 6:**
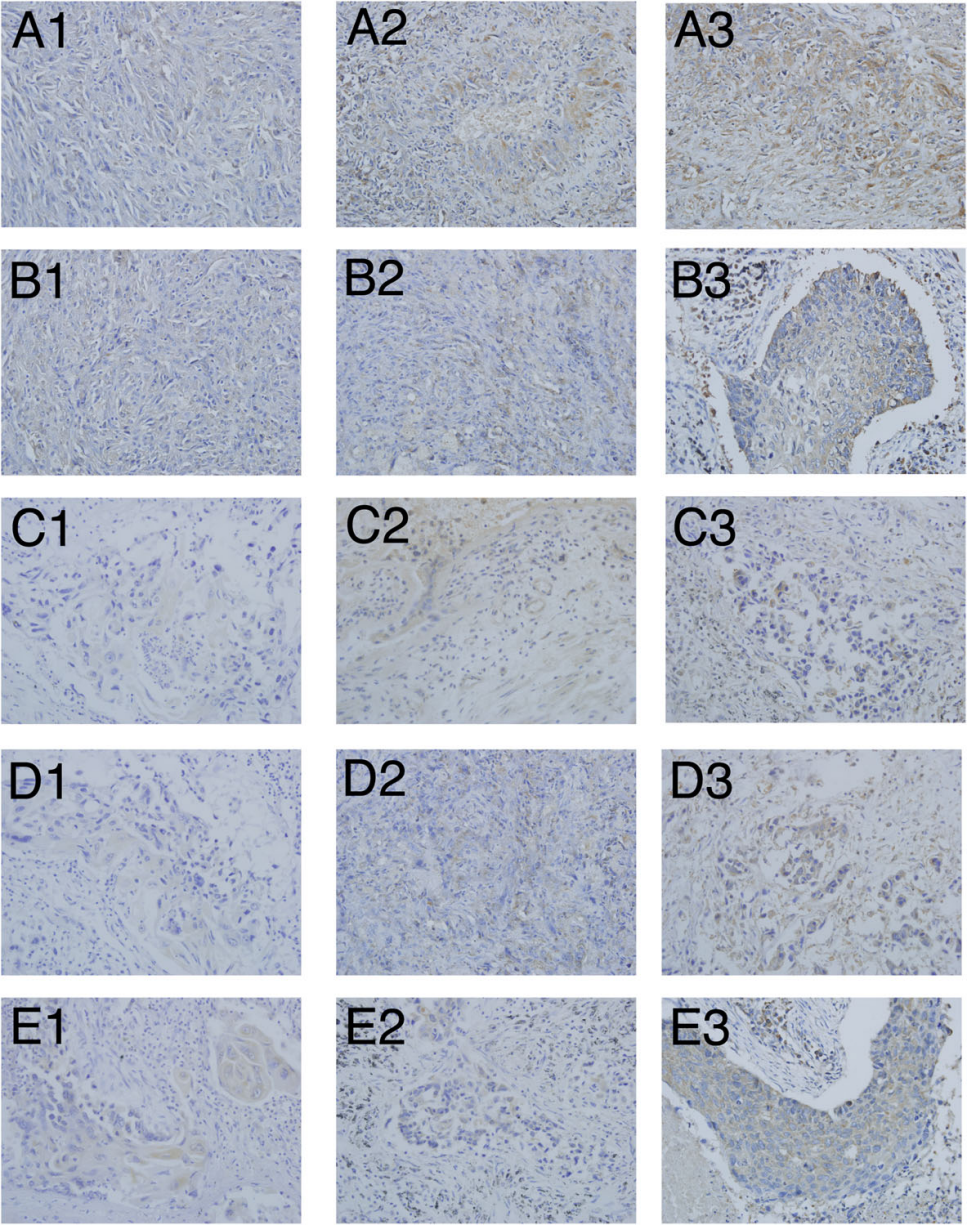
Immunohistochemical staining of FERMT2, FKBP3, SMAD9, GATA2 and IHITH4 protein in LUAD tissues (magnification, × 200). A1:weak expression of FERMT2;A2:moderate expression of FERMT2;A3:strong expression of FERMT2; B1:weak expression of FKBP3; B2:moderate expression of FKBP3;B3:strong expression of FKBP3;C1:weak expression of GATA2;C2:moderate expression of GATA2; C3:strong expression of GATA2;D1weak expression of IHITH4;D2:moderate expression of IHITH4;D3:strong expression of IHITH4; E1:weak expression of SMAD9;E2:moderate expression of SMAD9;E3:strong expression of SMAD9

In order to verify the clinical value of the model, we finally examined the expression of FERMT2, FKBP3, SMAD9, GATA2 and ITIH4 in 30 lung adenocarcinoma tissues by immunohistochemistry, considering the availability of antibodies. 86.67% (26/30) of LUAD patients tissue samples had positive expression of FERMT2, 83.33% (25/30) of FKBP3, 26.67% (8/30) of SMAD9, 23.33% (7/30) of GATA2 and 20.00% (6/30) of ITIH4 (Fig. 6). Based on the result of IHC of FERMT2, FKBP3, SMAD9, GATA2 and ITIH4, we divided the patients into 2 groups (negative group and positive group); the characteristics of each group are shown in Table 2.We found that the positive expression of FERMT2, FKBP3, SMAD9, GATA2 and ITIH4 had a correlation with the TNM stage, cellular differentiation and the lymph node metastasis (*p* < 0.05). No significant correlation was found with the age and sex (*p* > 0.05). We found that 83.33 and 91.67% of LUAD patients tissues in stage I-II (15/18) and stage III-IV (11/12) had positive expression of FERMT2(*P* < 0.05). 83.33% of LUAD patients tissues in stage I-II (15/18) and stage III-IV (10/12) had positive expression of FKBP3 (*P* > 0.05). Meanwhile, the positive rate of SMAD9, GATA2 and ITIH4 were 27.78% (5/18),16.67% (3/18) and 1.11% (2/18) in stage I-II and 25.00%(3/12),33.33%(4/12) and 33.33% (4/12) in stage III-IV(*P* < 0. 05). These were consistent with the results of our survival analysis: high levels of FERMT2, FKBP3 and low levels of SMAD9, ITIH4, GATA2 expression are associated with poor overall survival in LUAD.

**Table 2:** Baseline characteristics of patients

Clinical pathological characteristic	N	FERMT2		*P*	FKBP3		*P*	SMAD9		*P*	GATA2		*P*	ITIH4		*P*
		Positive	Negative		Positive	Negative		Positive	Negative		Positive	Negative		Positive	Negative	
Age (t/yr)							0.3321			0.5438			0.6755			0.6112
≥ 60	19	17	2	0.4130	17	2		5	14		4	15		4	15	
< 60	11	9	2		8	3		3	8		3	8		2	9	
Sex							0.5427			0.6472			0.7011			0.4328
Male	17	14	2	0.5052	15	2		4	13		4	13		3	14	
Female	13	12	1		10	3		2	11		3	10		3	10	
Histological differentiation							0.0382			0.0444			0.0311			0.0227
High or middle differentiation	10	9	1	0.0232	8	2		3	7		3	7		5	5	
Low or no differentiation	20	17	3		17	3		5	15		4	16		1	19	
TNM stage							0.0371			0.0319			0.0215			0.0333
I-II	18	15	3	0.0167	15	3		5	13		3	15		2	16	
III-IV	12	11	1		10	2		3	9		4	8		4	8	
Lymph node metastasis							0.0023			0.0034			0.0076			0.0041
No	14	11	3	0.0054	11	3		6	8		5	9		5	9	
Yes	16	15	1		14	2		2	14		3	14		1	15	

**Fig. 7:**
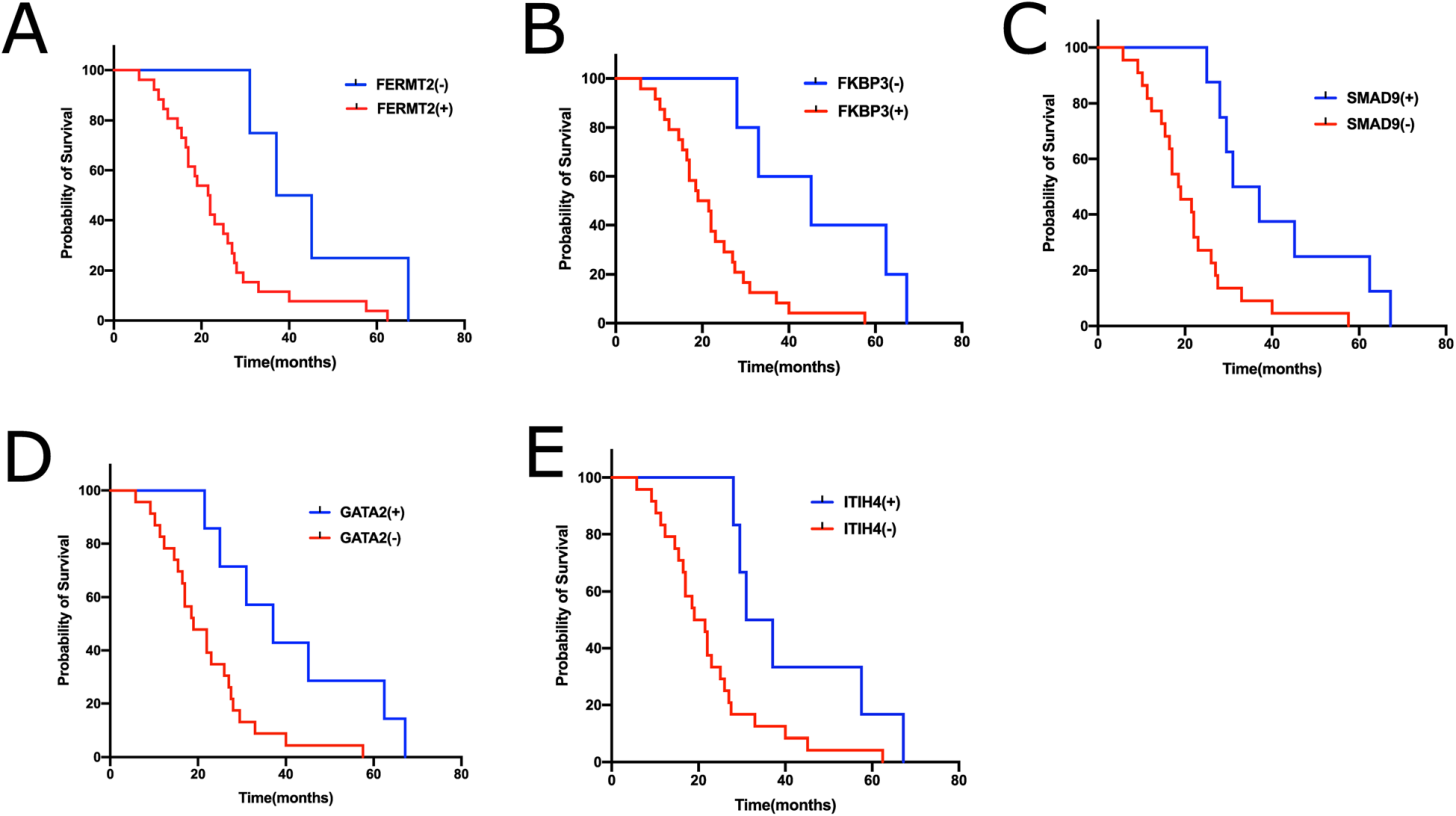
Effect of FERMT2, FKBP3, SMAD9, GATA2 and IHITH4 status on OS of LUAD patients. (**a**: Median survival time (MS) of LUAD patients with FERMT2 (−) and FERMT2 (+) was 41.4 months (95% CI, 37.1 to 45.2) and 21.70(95% CI,17.2 to 25.16), respectively, *P* = 0.0241. **b**: The MS of patients with FKBP3 (−) and FKBP3 (+) was 41.15 months (95% CI,36.23 to 46.72) and 20.25 (95% CI, 16.79 to 23.15), respectively, *P* = 0.0044. **c**: The MS of patients with SMAD9 (−) and SMAD9 (+) was 18.75 months (95% CI,13.15 to 23.38) and 34.05 (95% CI, 29.12 to 38.87), respectively, *P* = 0.0030. **d**: The MS of patients with GATA2 (−) and GATA2 (+) was 19.00 months (95% CI,14.47 to 24.13) and 37.10 (95% CI, 32.42 to 42.51), respectively, *P* = 0.0123. **e**: The MS of patients with ITIH4 (−) and ITIH4 (+) was 20.25 months (95% CI,15.14 to 25.42) and 34.05 (95% CI, 30.18 to 41.92), respectively, *P* = 0.0165)

Then Kaplan–Meier was performed to determine the effect of the immune related genes on prognosis of LUAD patients. Univariate Cox regression analysis demonstrated that the expression of FERMT2(HR = 5.084, 95% CI, 2.569 ~ 8.215), FKBP3(HR = 3.186, 95% CI, 2.279 ~ 7.945), SMAD9(HR = 0.791, 95%CI = 0.769 ~ 0.913), GATA2 (HR = 0.801, 95%CI = 0.744 ~ 0.952) and ITIH4 (HR = 0.776, 95%CI = 0.695 ~ 0.889) were significantly associated with overall survival (OS) (Fig. 7). Of note, The detailed coefficients of these five genes are 0.242851(FERMT2), 0.168033(FKBP3), − 0.00976(SMAD9), − 0.04737(GATA2) and − 0.0019 (ITIH4). The signs of those coefficients are consistent with the roles of the expression of those genes as revealed by survival analysis.

## Discussion

Adenocarcinoma is the most common pathological type of lung cancer with highly invasive and fatal. Most patients’ overall survival is less than 5 years whom were diagnosed at advanced stage [26]. Existing treatments extend the survival of part of patients with lung adenocarcinoma, but the overall curative effect is not so good, especially in the advanced cases [27, 28]. The shortage of effective prognostic biomarkers to guide therapy is one of the reasons for the poor prognosis [29]. Therefore, there is a need to construct an efficient prognostic model to develop individualized treatment plans for patients and improve the prognosis of LUAD.

Current studies have found that the development of cancer are not only dependent on tumor cell characteristics but are also affected by the interaction with infiltrated immunocytes [30, 31]. The tumors with higher immune cells and mediators proportion were proved to be more effective to the immune treatment [32]. There is mounting evidence supporting that the immunogenomics and immune microenvironment play an important role in cancer [33, 34]. As an example, at the levels of DNA, RNA and the epigenome, Rosenthal et al. has observed the signs of immunologic sculpting, immunoediting, and immune escape [35]. These studies provide the clues for our research toward DEIGs. In our study, the DEIGs were identified by the bioinformatics analysis with TCGA datasets, we found that the inflammatory pathway was an inseparable aspect of tumor development. Similar results were found in other studies [36–38].

Four prediction models were built with lasso regression using distinct lists of immune related genes. Model 4 which contains 33 prognosis DEIGs performed best in prognostic predictions, and correlated with tumor burden, tumor stage and metastasis. Among those prognosis-specific immune related genes, 14(e.g., CCT6A, EFNB2, FKBP3, FERMT2, SMAD9, GATA2, PFKP, PKP2, PTPRH, CCL20, SLC7A5, DDIT4, LY6K, ITIH4) have been demonstrated to be participate in the the pathogenesis of cancer or reported to be significant predictors of survival [39–46]. This implies that our analysis has certain theoretic value. The remaining genes which have not been reported could serve as new potential biomarkers of LUAD.

On one hand, the coef of FERMT2 and FKBP3 were highest, and the expression of FERMT2, FKBP3, SMAD9, GATA2 and ITIH4 in the tissues of LUAD patients and their correlation with patient survival have not been studied. On the other hand, considering the availability of antibodies, we finally examined the expression of FERMT2, FKBP3, SMAD9, GATA2 and ITIH4 in 30 LUAD tissues by immunohistochemistry. Previous studies showed that FERMT2 highly expressed in NSCLC, esophageal squamous cancer, breast cancer, cholangiocarcinoma and pancreatic cancer, and can affect the migration ability of tumor cells and disease progression [47–49]. Guo et al. found the expression of FERMT2 is closely correlated with the tumor clinical stage of lung cancer [50]. Our findings concordant with these results. It is hypothesized that FERMT2 may have effects on tumor immunity through interactions with integrin-like protein. A large number of studies have proved that HDACs are involved in regulating the innate and adaptive immune processes of the body [51]. FKBP3 which is a member of FK506-binding proteins, could promote proliferation of lung cancer cells through regulating Sp1/HDAC2/p27 [52], we assumed that its immunoregulation effects could be related to HDAC2 [53]. Meanwhile, there is plenty of evidence that SMAD9, ITIH4 and GATA2 have close connection with the initiation, progression and prognosis of various malignancies including lung cancer [54–57]. SMAD9 is located on chromosome 13q13.3 and encodes a protein that is a member of the SMAD family, which is a crucial pathway for the TGF-β transcription factor family [58]. It was found that SMAD9 may be regulated by methylation, phosphorylation and dephosphorylation in the occurrence and development of lung cancer [59]. Previous studies suggested that GATA2 is important for survival and growth of NSCLC cells with mutations in KRAS and other oncogenes on the RTK/RAS pathway. The deletion of GATA2 reduces survival of KRAS mutant NSCLC cells significantly inhibit the development of NSCLC [60]. In addition, recent study reported that GATA2 is sufficient to drive PD-L1 and PD-L2 expression and is necessary for PD-L2 expression. It was reported that cytokines, such as IL-6, TNF-α, IL-10 and lipopolysaccharide (LPS) influence the expression of ITIH4. ITIH4, as an inflammation biomarker may participate in immune regulation through JAK/STAT [61].

In our study, we also revealed that the expression of FERMT2, FKBP3, SMAD9, ITIH4 and GATA2 are independent prognostic factors, furthermore, high levels of FERMT2, FKBP3 and low levels of SMAD9, ITIH4, GATA2 expression are associated with poor overall survival in LUAD.

Combined with the TCGA database analysis and literature reports in this study, we speculated that the expression of these genes that influence tumor prognosis are significantly correlated with multiple cytokine pathways and immunity correlation reaction.

As we know, the tumor immune microenvironment was composed of various infiltrating immune cells including T cells, B cells, natural killer cells, dendritic cells, myeloid-derived suppressor cells, neutrophils, and macrophages [62, 63]. Lots of studies have demonstrated the relationship between the tumor-infiltrating immune cells and tumor growth, metastasis or angiogenesis of lung cancer [64–66]. These reports are in line with our results. We found that the risk score of our model was inversely related to the infiltration of various immune cells, as well as the markers of B cell, CD4+ T cell, CD8+ T cell and dendritic cell. These results indicated that the high-risk patients’ infiltration levels of immune cells might be lower, suggesting that the abnormal expression of immune genes can lead to the disorder of tumor immune microenvironment, and then participate in the occurrence, development, invasion and metastasis of LUAD.

## Conclusions

In this study, we constructed 4 models to predict the prognosis of patients with LUAD, and proposed an optimal prognostic model, our preliminary results hint at the correlation between immune related genes and the prognosis of LUAD. However, the further research about the mechanisms of the DEIGs modulate the progression of LUAD is needed.

## Supplementary Information

**Additional file 1: Table S1.** A total of 436 differentially expressed genes were identified. **Figure S1.** PPI network inferred by STRING using 436 differentially expressed immune genes with *p*.value < 0.05 in cox test. Hub genes were identified by cytoscape. Shown is cluster1 with 31 genes. **Figure S2.** Mutation landscape of 436 target genes (top50) in 569 tumors. **Figure S3.** The coefficients for the genes retained in model 4. **Table S2.** The list of coefficients for genes retained in model 4. **Figure S4.** Identification of an immune signature predicting prognosis risk of patients in LUAD using each models (A1,B1,C1: A cutoff of risk factor for modle 1, modle 2, modle 3; A2,B2,C2: Survival analysis of the training dataset for modle 1, modle 2, modle 3; A3,B3,C3: Survival analysis in the testing data for modle 1, modle 2, modle 3). **Figure S5.** Relationships between the risk score and CD8+ T cell markers. **Figure S6.** Relationships between the risk score and CD4+ T cell markers. **Figure S7.** Relationships between the risk score and B cell markers. **Figure S8.** Relationships between the risk score and dendritic cell markers.

## Data Availability

All data generated or analyzed during this study are included in this published article and its Supplementary Information files. The datasets generated and/or analyzed during the current study are available in the TCGA repository (https://portal.gdc.cancer.gov/). The immune-related genes were derived from InnateDB (https://www.innatedb.com/). While the estimated infiltration abundance of immune cells of LUAD samples were obtained by TIMER (https://cistrome.shinyapps.io/timer/).

## Abbreviations

TCGA: The Cancer Genome Atlas
NSCLC: Non small-cell lung cancer
LUAD: Lung adenocarcinoma
DEIGs: Differentially expressed immune genes
TME: Tumor microenvironment
TMB: Tumor mutational burden
MMR: Mismatch repair
GO: Gene Ontology
KEGG: Kyoto Encyclopedia of Genes and Genomes
PPI: Protein-protein interaction
ROC: Receiver operating characteristic curve
IHC: Immunohistochemistry
HRP: Horseradish peroxidase
IRS: Immunoreactivity score
