# Supplementary material for: Construction of the optimization prognostic model based on differentially expressed immune genes of lung adenocarcinoma
Source: BMC Cancer. 2021 Mar 1;21:213. doi: 10.1186/s12885-021-07911-8 (PMC7923649; doi:10.1186/s12885-021-07911-8)

Table S1: A total of 436 differentially expressed genes were identified

| Gene | HR | 95%CI | p value |
| --- | --- | --- | --- |
| A2M | **0.87** | **(0.76-0.98)** | **0.026** |
| ACHE | 0.92 | (0.85-0.99) | 0.021 |
| ACP5 | 0.83 | (0.72-0.97) | 0.018 |
| ACTL6A | 1.4 | (1.1-1.7) | 0.0046 |
| ACTN2 | 0.88 | (0.81-0.96) | 0.0039 |
| ADA | 1.2 | (1.1-1.4) | 0.0024 |
| ADRA1A | 0.9 | (0.83-0.97) | 0.0072 |
| ADRB1 | 0.92 | (0.86-0.98) | 0.012 |
| ADRB2 | 0.85 | (0.77-0.94) | 0.0018 |
| ACAN | 1.1 | (1-1.2) | 0.023 |
| AGER | 0.91 | (0.86-0.97) | 0.0033 |
| ALDH2 | 0.8 | (0.7-0.92) | 0.0018 |
| ALOX5AP | 0.9 | (0.81-0.99) | 0.039 |
| ALOX15 | 0.93 | (0.88-0.98) | 0.007 |
| APBB1 | 0.85 | (0.73-0.99) | 0.043 |
| BIRC5 | 1.2 | (1.1-1.3) | 0.00029 |
| AQP4 | 0.93 | (0.9-0.98) | 0.0017 |
| ART4 | 0.92 | (0.85-1) | 0.043 |
| ASPH | 1.2 | (1.1-1.4) | 0.0024 |
| ATIC | 1.6 | (1.2-2.1) | 0.0015 |
| TNFRSF17 | 0.91 | (0.85-0.98) | 0.013 |
| BIK | 1.1 | (1-1.3) | 0.047 |
| BLM | 1.3 | (1.1-1.5) | 0.00025 |
| BRCA1 | 1.2 | (1.1-1.4) | 0.004 |
| BRCA2 | 1.2 | (1.1-1.4) | 0.006 |
| BTK | 0.84 | (0.75-0.94) | 0.0018 |
| BTN1A1 | 0.89 | (0.82-0.98) | 0.019 |
| BUB1 | 1.2 | (1.1-1.4) | 0.0005 |
| BUB1B | 1.2 | (1.1-1.4) | 0.00017 |
| C6 | 0.92 | (0.88-0.97) | 0.0034 |
| C7 | 0.93 | (0.88-0.99) | 0.022 |
| C8B | 0.94 | (0.89-0.99) | 0.012 |
| CA4 | 0.94 | (0.9-0.99) | 0.022 |
| CACNB4 | 0.84 | (0.76-0.94) | 0.0022 |
| CAMP | 0.91 | (0.85-0.97) | 0.0059 |
| CAT | 0.76 | (0.63-0.91) | 0.0033 |
| CBFA2T3 | 0.78 | (0.69-0.88) | 0.000041 |
| CCNE1 | 1.2 | (1-1.3) | 0.0037 |
| CCT6A | 1.5 | (1.3-1.8) | 0.00000098 |
| CD1A | 0.94 | (0.89-0.99) | 0.019 |
| CD27 | 0.87 | (0.79-0.97) | 0.0098 |
| CD33 | 0.89 | (0.8-0.99) | 0.038 |
| CD37 | 0.85 | (0.75-0.95) | 0.0067 |
| CD69 | 0.87 | (0.78-0.96) | 0.0072 |
| CD79A | 0.89 | (0.82-0.96) | 0.0025 |
| CDA | 1.1 | (1-1.1) | 0.045 |
| CDK1 | 1.3 | (1.1-1.4) | 0.000065 |
| CDC25A | 1.2 | (1.1-1.4) | 0.00043 |
| CDH15 | 0.93 | (0.87-1) | 0.039 |
| CDH17 | 1.1 | (1-1.1) | 0.0022 |
| CD52 | 0.84 | (0.74-0.94) | 0.0027 |
| CFTR | 0.92 | (0.87-0.96) | 0.00082 |
| CEACAM8 | 0.91 | (0.86-0.98) | 0.0075 |
| ABCC2 | 1.1 | (1-1.1) | 0.000032 |
| COL1A1 | 1.1 | (1-1.2) | 0.026 |
| COL1A2 | 1.1 | (1-1.3) | 0.024 |
| COL4A3 | 0.9 | (0.84-0.95) | 0.00065 |
| COL5A1 | 1.1 | (1-1.3) | 0.019 |
| COL7A1 | 1.1 | (1-1.1) | 0.014 |
| COL9A2 | 0.9 | (0.83-0.98) | 0.016 |
| COL11A1 | 1.1 | (1-1.1) | 0.013 |
| COL13A1 | 0.88 | (0.79-0.97) | 0.01 |
| MAP3K8 | 0.77 | (0.64-0.92) | 0.0044 |
| CPA3 | 0.91 | (0.85-0.98) | 0.0074 |
| VCAN | 1.1 | (1-1.2) | 0.03 |
| CTLA4 | 0.88 | (0.8-0.98) | 0.016 |
| CTSG | 0.92 | (0.86-0.97) | 0.0041 |
| CTSL2 | 1.2 | (1.1-1.2) | 0.00025 |
| CX3CR1 | 0.88 | (0.81-0.96) | 0.0027 |
| DNASE1 | 1.2 | (1-1.4) | 0.028 |
| DNASE1L3 | 0.91 | (0.85-0.97) | 0.0038 |
| DPP4 | 0.93 | (0.86-0.99) | 0.035 |
| DSC1 | 1.2 | (1.1-1.3) | 0.00066 |
| DSG2 | 1.4 | (1.2-1.6) | 0.000027 |
| DSP | 1.1 | (1-1.2) | 0.021 |
| ECT2 | 1.4 | (1.2-1.6) | 0.0000079 |
| EDN3 | 0.9 | (0.84-0.97) | 0.007 |
| EFNB2 | 1.2 | (1.1-1.4) | 0.0045 |
| EGR2 | 0.88 | (0.78-1) | 0.043 |
| ENO1 | 1.4 | (1.1-1.8) | 0.0057 |
| EREG | 1.1 | (1-1.1) | 0.0011 |
| ETV5 | 0.83 | (0.71-0.96) | 0.013 |
| EVI2B | 0.86 | (0.76-0.98) | 0.021 |
| F2RL1 | 1.2 | (1.1-1.3) | 0.002 |
| F10 | 0.88 | (0.79-0.98) | 0.025 |
| F12 | 1.2 | (1-1.3) | 0.006 |
| FANCA | 1.2 | (1-1.4) | 0.02 |
| FANCD2 | 1.3 | (1.1-1.6) | 0.0027 |
| FANCB | 1.2 | (1-1.3) | 0.017 |
| FAT1 | 1.2 | (1-1.3) | 0.018 |
| FBP1 | 0.78 | (0.69-0.89) | 0.00014 |
| MS4A2 | 0.9 | (0.83-0.96) | 0.0032 |
| FCN1 | 0.91 | (0.84-0.99) | 0.032 |
| FEN1 | 1.4 | (1.2-1.7) | 0.00014 |
| FGF10 | 0.86 | (0.77-0.96) | 0.0086 |
| FGFR2 | 0.89 | (0.81-0.98) | 0.021 |
| FGR | 0.87 | (0.77-0.99) | 0.03 |
| FIGF | 0.92 | (0.87-0.97) | 0.0028 |
| FKBP3 | 1.3 | (1.1-1.7) | 0.0054 |
| FKBP4 | 1.5 | (1.3-1.8) | 0.0000053 |
| DARC | 0.93 | (0.88-1) | 0.043 |
| GAP43 | 1.1 | (1-1.1) | 0.031 |
| GARS | 1.4 | (1.1-1.8) | 0.0016 |
| GATA2 | 0.86 | (0.76-0.98) | 0.024 |
| GPC3 | 0.89 | (0.82-0.97) | 0.01 |
| GCLM | 1.1 | (1-1.3) | 0.037 |
| GPI | 1.5 | (1.2-1.8) | 0.00017 |
| GPR17 | 0.9 | (0.82-0.99) | 0.03 |
| GRIK2 | 1.1 | (1-1.2) | 0.006 |
| HIST1H2BD | 1.2 | (1-1.3) | 0.023 |
| HABP2 | 0.91 | (0.87-0.96) | 0.00019 |
| HAS1 | 1.1 | (1-1.1) | 0.044 |
| HELLS | 1.2 | (1.1-1.4) | 0.0044 |
| NCKAP1L | 0.89 | (0.8-1) | 0.048 |
| HLF | 0.87 | (0.81-0.94) | 0.00012 |
| HMGA1 | 1.3 | (1.2-1.5) | 0.00003 |
| HMMR | 1.3 | (1.2-1.5) | 0.0000059 |
| HNF4A | 1.1 | (1-1.1) | 0.032 |
| HPRT1 | 1.2 | (1-1.5) | 0.031 |
| HSPD1 | 1.5 | (1.2-1.8) | 0.00047 |
| HSPE1 | 1.2 | (1-1.5) | 0.044 |
| ICAM4 | 0.92 | (0.85-0.99) | 0.027 |
| IGFBP3 | 1.1 | (1-1.3) | 0.027 |
| IL3RA | 0.84 | (0.72-0.98) | 0.031 |
| IL5RA | 0.92 | (0.86-0.99) | 0.02 |
| IL7R | 0.9 | (0.83-0.98) | 0.016 |
| IL11 | 1.1 | (1-1.2) | 0.004 |
| IL11RA | 0.74 | (0.61-0.89) | 0.0012 |
| IL12B | 0.9 | (0.82-0.99) | 0.033 |
| INCENP | 1.3 | (1.1-1.5) | 0.0022 |
| ITGA2 | 1.1 | (1-1.2) | 0.046 |
| ITGAL | 0.82 | (0.73-0.93) | 0.0018 |
| ITGB4 | 1.1 | (1-1.3) | 0.0063 |
| ITIH4 | 0.88 | (0.81-0.96) | 0.0025 |
| KAL1 | 0.85 | (0.77-0.93) | 0.00082 |
| KCNJ15 | 0.92 | (0.85-0.99) | 0.026 |
| KPNA2 | 1.4 | (1.2-1.6) | 0.000035 |
| KRT18 | 1.5 | (1.2-1.7) | 0.000013 |
| AFF3 | 0.91 | (0.84-0.98) | 0.013 |
| LCP1 | 0.88 | (0.77-0.99) | 0.037 |
| LDHA | 1.7 | (1.4-2.1) | 0.00000016 |
| LIFR | 0.8 | (0.71-0.91) | 0.00049 |
| LMNB1 | 1.3 | (1.1-1.5) | 0.00049 |
| LOXL2 | 1.3 | (1.1-1.4) | 0.000013 |
| LPL | 0.92 | (0.85-0.99) | 0.021 |
| LSAMP | 0.88 | (0.78-0.98) | 0.018 |
| LTC4S | 0.85 | (0.77-0.94) | 0.0013 |
| MAD2L1 | 1.3 | (1.1-1.4) | 0.00011 |
| SMAD9 | 0.89 | (0.81-0.97) | 0.011 |
| MAL | 0.85 | (0.77-0.93) | 0.00051 |
| MAOB | 0.86 | (0.78-0.95) | 0.0029 |
| MC1R | 1.2 | (1.1-1.4) | 0.0036 |
| MCM7 | 1.2 | (1-1.4) | 0.044 |
| MDFI | 1.2 | (1.1-1.3) | 0.00077 |
| MET | 1.1 | (1-1.2) | 0.025 |
| MFAP4 | 0.87 | (0.8-0.96) | 0.004 |
| MFI2 | 1.2 | (1.1-1.3) | 0.000031 |
| MFNG | 0.78 | (0.66-0.93) | 0.0047 |
| MIF | 1.2 | (1.1-1.4) | 0.0052 |
| MKI67 | 1.3 | (1.1-1.4) | 0.000076 |
| MNDA | 0.89 | (0.8-0.99) | 0.026 |
| MST1 | 0.88 | (0.79-0.98) | 0.019 |
| MSX2 | 1.1 | (1-1.2) | 0.015 |
| MUSK | 0.89 | (0.82-0.96) | 0.0027 |
| NEDD9 | 0.86 | (0.75-0.97) | 0.019 |
| NELL2 | 0.9 | (0.83-0.98) | 0.013 |
| NFATC1 | 0.85 | (0.73-0.99) | 0.037 |
| NME1 | 1.2 | (1-1.4) | 0.034 |
| NRAP | 0.9 | (0.82-0.98) | 0.013 |
| NT5E | 1.1 | (1-1.2) | 0.011 |
| OAS1 | 1.2 | (1-1.3) | 0.025 |
| P2RX1 | 0.82 | (0.73-0.91) | 0.00019 |
| FURIN | 1.2 | (1.1-1.4) | 0.00065 |
| PAFAH1B3 | 1.2 | (1-1.4) | 0.032 |
| PAK1 | 1.3 | (1-1.7) | 0.02 |
| PCDH7 | 1.2 | (1.1-1.3) | 0.00022 |
| PCNA | 1.2 | (1-1.5) | 0.022 |
| PDGFB | 1.2 | (1.1-1.4) | 0.0039 |
| PFKP | 1.3 | (1.1-1.5) | 0.00012 |
| PGM5 | 0.87 | (0.79-0.96) | 0.0043 |
| PHKA1 | 1.2 | (1-1.5) | 0.026 |
| PIK3R1 | 0.81 | (0.66-0.99) | 0.039 |
| PITX1 | 1.1 | (1-1.1) | 0.026 |
| PKP2 | 1.2 | (1.1-1.3) | 0.000012 |
| PLA2G1B | 0.92 | (0.88-0.97) | 0.0016 |
| PMAIP1 | 1.1 | (1-1.3) | 0.031 |
| SEPT4 | 0.8 | (0.68-0.95) | 0.0091 |
| PRKCE | 0.73 | (0.6-0.9) | 0.003 |
| PRKCH | 0.75 | (0.58-0.97) | 0.026 |
| PRKDC | 1.3 | (1.1-1.5) | 0.0082 |
| PTGDS | 0.85 | (0.77-0.93) | 0.00048 |
| PTGFRN | 1.3 | (1.1-1.6) | 0.0044 |
| PTHLH | 1.1 | (1-1.2) | 0.046 |
| PTPRH | 1.1 | (1.1-1.2) | 0.00032 |
| PTPRO | 0.9 | (0.81-1) | 0.045 |
| PTX3 | 1.1 | (1-1.2) | 0.027 |
| RAB3B | 1.1 | (1-1.1) | 0.014 |
| RAB27B | 1.1 | (1-1.2) | 0.0099 |
| RASGRF1 | 0.91 | (0.85-0.97) | 0.0035 |
| GRK1 | 0.91 | (0.85-0.99) | 0.02 |
| ROBO2 | 0.91 | (0.85-0.98) | 0.011 |
| RORA | 0.84 | (0.72-0.98) | 0.023 |
| MRPS12 | 1.3 | (1-1.5) | 0.016 |
| RS1 | 0.9 | (0.83-0.98) | 0.016 |
| RTN1 | 0.89 | (0.81-0.98) | 0.015 |
| S100P | 1.1 | (1-1.1) | 0.0085 |
| CCL20 | 1.1 | (1-1.2) | 0.011 |
| SFRP5 | 0.95 | (0.9-1) | 0.049 |
| SFTPD | 0.91 | (0.86-0.96) | 0.00037 |
| SIX1 | 0.92 | (0.85-1) | 0.04 |
| SLA | 0.87 | (0.77-0.99) | 0.04 |
| SLC5A4 | 0.89 | (0.81-0.98) | 0.014 |
| SLC6A8 | 1.1 | (1-1.3) | 0.02 |
| SLC14A1 | 0.87 | (0.79-0.96) | 0.0042 |
| SLC15A2 | 0.86 | (0.79-0.94) | 0.0012 |
| FSCN1 | 1.3 | (1.1-1.4) | 0.000015 |
| SNRPA1 | 1.4 | (1.1-1.7) | 0.0019 |
| SNRPE | 1.4 | (1.1-1.8) | 0.0058 |
| SPOCK1 | 1.1 | (1.1-1.2) | 0.00053 |
| SRM | 1.3 | (1-1.6) | 0.048 |
| SRPK1 | 1.5 | (1.1-1.9) | 0.0031 |
| SSR4 | 0.83 | (0.7-0.99) | 0.037 |
| SULT1A1 | 0.84 | (0.74-0.96) | 0.0078 |
| TCF21 | 0.88 | (0.81-0.96) | 0.0044 |
| TCN1 | 1.1 | (1-1.1) | 0.0028 |
| LEFTY2 | 0.92 | (0.86-0.99) | 0.018 |
| KLF10 | 1.4 | (1.1-1.8) | 0.0044 |
| TOP2A | 1.2 | (1.1-1.3) | 0.0015 |
| TPBG | 1.3 | (1.1-1.5) | 0.0036 |
| TPI1 | 1.5 | (1.2-1.9) | 0.00023 |
| CCT3 | 1.5 | (1.2-1.9) | 0.0015 |
| TUBA4A | 1.2 | (1-1.4) | 0.015 |
| TWIST1 | 1.1 | (1-1.2) | 0.05 |
| TXNRD1 | 1.2 | (1-1.3) | 0.0033 |
| UPK1B | 1.1 | (1-1.1) | 0.00046 |
| SCGB1A1 | 0.96 | (0.93-0.99) | 0.022 |
| VIPR1 | 0.84 | (0.76-0.92) | 0.00011 |
| BTG2 | 0.79 | (0.7-0.9) | 0.00025 |
| LST1 | 0.88 | (0.79-0.99) | 0.03 |
| AIMP2 | 1.3 | (1.1-1.6) | 0.014 |
| ADAM12 | 1.1 | (1-1.2) | 0.0048 |
| SLC7A5 | 1.2 | (1.1-1.3) | 0.0046 |
| FKBP6 | 0.85 | (0.78-0.93) | 0.00045 |
| GPR65 | 0.87 | (0.76-0.98) | 0.025 |
| SEMA7A | 1.2 | (1-1.3) | 0.016 |
| PPFIBP1 | 1.3 | (1.1-1.6) | 0.0037 |
| KCNAB2 | 0.85 | (0.74-0.98) | 0.028 |
| CDC14A | 0.84 | (0.7-1) | 0.05 |
| SCARF1 | 0.77 | (0.66-0.9) | 0.001 |
| TNFSF11 | 1.1 | (1-1.1) | 0.048 |
| KCNK5 | 0.87 | (0.79-0.95) | 0.0025 |
| S1PR4 | 0.84 | (0.73-0.96) | 0.011 |
| B3GALNT1 | 1.2 | (1-1.4) | 0.018 |
| TNFSF13 | 0.79 | (0.68-0.93) | 0.0033 |
| TNFSF12 | 0.76 | (0.63-0.93) | 0.0073 |
| CDK5R1 | 1.3 | (1.1-1.4) | 0.00088 |
| IER3 | 1.2 | (1-1.3) | 0.017 |
| WASF1 | 1.2 | (1-1.4) | 0.011 |
| KYNU | 1.2 | (1.1-1.3) | 0.00016 |
| DOK2 | 0.88 | (0.78-1) | 0.046 |
| PRC1 | 1.3 | (1.2-1.5) | 0.0000096 |
| CLDN2 | 0.93 | (0.89-0.98) | 0.0023 |
| CCNB2 | 1.3 | (1.1-1.4) | 0.000099 |
| EXO1 | 1.3 | (1.1-1.4) | 0.0000066 |
| AURKB | 1.2 | (1.1-1.3) | 0.0014 |
| CD83 | 0.83 | (0.72-0.96) | 0.014 |
| KL | 0.86 | (0.78-0.95) | 0.0038 |
| CD101 | 0.85 | (0.75-0.97) | 0.014 |
| LPXN | 0.8 | (0.67-0.95) | 0.013 |
| LY86 | 0.88 | (0.79-0.99) | 0.027 |
| ARHGEF6 | 0.82 | (0.71-0.94) | 0.0059 |
| SH3BP5 | 0.75 | (0.64-0.89) | 0.00085 |
| GDF15 | 0.89 | (0.81-0.97) | 0.0077 |
| MTL5 | 1.2 | (1.1-1.3) | 0.00011 |
| RIMS2 | 1.1 | (1-1.1) | 0.003 |
| ESPL1 | 1.2 | (1.1-1.3) | 0.00063 |
| DOCK4 | 0.8 | (0.67-0.94) | 0.008 |
| SPOCK2 | 0.88 | (0.77-1) | 0.048 |
| P2RY14 | 0.84 | (0.74-0.95) | 0.0068 |
| CD302 | 0.77 | (0.67-0.88) | 0.00019 |
| DLEC1 | 0.91 | (0.84-0.97) | 0.0081 |
| BCL2L10 | 1.1 | (1-1.2) | 0.0071 |
| TROAP | 1.1 | (1-1.3) | 0.0026 |
| TSPAN32 | 0.83 | (0.75-0.92) | 0.00029 |
| PPIF | 1.3 | (1-1.6) | 0.035 |
| SORBS3 | 0.81 | (0.66-1) | 0.049 |
| RASGRP2 | 0.83 | (0.75-0.93) | 0.001 |
| SEMA3A | 1.1 | (1-1.2) | 0.012 |
| DLC1 | 0.89 | (0.81-0.98) | 0.022 |
| TACC3 | 1.4 | (1.2-1.6) | 0.000083 |
| SEMA4B | 1.3 | (1.1-1.5) | 0.0017 |
| FBLN5 | 0.88 | (0.77-1) | 0.05 |
| CIB2 | 1.1 | (1-1.3) | 0.018 |
| POSTN | 1.1 | (1-1.3) | 0.013 |
| IGF2BP3 | 1.1 | (1-1.2) | 0.0011 |
| PLK4 | 1.3 | (1.1-1.4) | 0.00066 |
| WASF3 | 0.88 | (0.79-0.99) | 0.036 |
| NOXA1 | 0.9 | (0.81-1) | 0.048 |
| CKAP4 | 1.4 | (1.2-1.8) | 0.00034 |
| FERMT2 | 1.3 | (1-1.6) | 0.027 |
| LILRA1 | 0.88 | (0.8-0.97) | 0.011 |
| RAPGEF4 | 0.86 | (0.75-1) | 0.045 |
| ADAMTS8 | 0.91 | (0.85-0.97) | 0.005 |
| CD160 | 0.8 | (0.69-0.92) | 0.0019 |
| PKP3 | 1.3 | (1.1-1.5) | 0.011 |
| MRPL3 | 1.3 | (1-1.8) | 0.033 |
| CBX3 | 1.4 | (1.1-1.8) | 0.0058 |
| PTGR1 | 1.1 | (1-1.3) | 0.029 |
| TPX2 | 1.3 | (1.1-1.4) | 0.000016 |
| IGSF9B | 0.92 | (0.87-0.98) | 0.0099 |
| NFASC | 0.9 | (0.81-1) | 0.048 |
| ARC | 0.92 | (0.85-1) | 0.05 |
| SEC61G | 1.5 | (1.2-1.8) | 0.000051 |
| CBX7 | 0.74 | (0.62-0.87) | 0.00035 |
| VSIG2 | 0.91 | (0.86-0.96) | 0.00028 |
| DAPK2 | 0.77 | (0.67-0.87) | 0.000048 |
| CADM1 | 0.9 | (0.82-0.99) | 0.034 |
| FLRT1 | 1.1 | (1-1.2) | 0.045 |
| NGEF | 1.1 | (1-1.2) | 0.0027 |
| METTL7A | 0.74 | (0.64-0.85) | 0.000034 |
| POC1A | 1.3 | (1.1-1.5) | 0.0037 |
| LATS2 | 1.3 | (1.1-1.7) | 0.017 |
| MYEOV | 1 | (1-1.1) | 0.05 |
| GREM1 | 1.1 | (1-1.1) | 0.033 |
| CKAP2 | 1.3 | (1.1-1.5) | 0.012 |
| HAVCR1 | 1 | (1-1.1) | 0.045 |
| PABPC1 | 1.3 | (1.1-1.6) | 0.0088 |
| SESN1 | 0.82 | (0.67-1) | 0.049 |
| CNTN6 | 0.95 | (0.89-1) | 0.046 |
| HPGDS | 0.87 | (0.8-0.94) | 0.00047 |
| PCDH11X | 0.92 | (0.84-1) | 0.046 |
| BZW2 | 1.4 | (1.1-1.7) | 0.0013 |
| MRPL15 | 1.3 | (1.1-1.6) | 0.0018 |
| C11orf21 | 0.81 | (0.73-0.91) | 0.00016 |
| RACGAP1 | 1.3 | (1.1-1.5) | 0.00046 |
| ERO1L | 1.4 | (1.2-1.6) | 0.00000027 |
| CD207 | 0.93 | (0.89-0.98) | 0.0076 |
| SIDT2 | 0.74 | (0.58-0.94) | 0.015 |
| ANGPTL4 | 1.2 | (1.1-1.2) | 0.0003 |
| NUSAP1 | 1.3 | (1.1-1.4) | 0.00035 |
| CLDN18 | 0.95 | (0.92-0.99) | 0.015 |
| TM6SF1 | 0.85 | (0.74-0.97) | 0.019 |
| IL20RB | 1.1 | (1-1.2) | 0.0024 |
| DUOX1 | 0.93 | (0.87-1) | 0.05 |
| ANLN | 1.3 | (1.2-1.5) | 0.000000089 |
| DDIT4 | 1.2 | (1.1-1.4) | 0.0014 |
| LY6K | 1.1 | (1-1.1) | 0.0006 |
| ERCC6L | 1.3 | (1.1-1.4) | 0.00041 |
| ZWILCH | 1.4 | (1.2-1.8) | 0.00054 |
| HEATR1 | 1.5 | (1.1-2) | 0.0031 |
| FANCI | 1.4 | (1.2-1.6) | 0.00013 |
| NEIL3 | 1.2 | (1.1-1.3) | 0.000023 |
| DRAM1 | 0.85 | (0.75-0.96) | 0.0094 |
| FERMT1 | 1.2 | (1-1.3) | 0.016 |
| NLRP2 | 0.95 | (0.9-1) | 0.047 |
| LIMS2 | 0.84 | (0.72-0.98) | 0.023 |
| PRR11 | 1.2 | (1.1-1.3) | 0.0017 |
| C20orf24 | 1.3 | (1.1-1.6) | 0.0062 |
| PCDHGA1 | 1.1 | (1-1.2) | 0.042 |
| CRTAM | 0.89 | (0.8-0.99) | 0.026 |
| CPXM1 | 1.1 | (1-1.3) | 0.027 |
| CASS4 | 0.85 | (0.76-0.94) | 0.0027 |
| AMIGO1 | 0.83 | (0.72-0.95) | 0.0074 |
| EPB41L5 | 0.77 | (0.65-0.91) | 0.0028 |
| MS4A7 | 0.87 | (0.77-0.98) | 0.018 |
| NLRC4 | 0.83 | (0.73-0.95) | 0.0079 |
| ALOXE3 | 1.1 | (1-1.2) | 0.0078 |
| HAPLN2 | 1.2 | (1-1.3) | 0.0053 |
| PERP | 1.4 | (1.2-1.6) | 0.000086 |
| SMOC1 | 1.1 | (1-1.1) | 0.046 |
| CENPK | 1.2 | (1.1-1.4) | 0.00029 |
| NFKBIZ | 0.88 | (0.78-1) | 0.043 |
| CDH24 | 1.2 | (1.1-1.4) | 0.0048 |
| P2RY12 | 0.91 | (0.85-0.98) | 0.016 |
| MRPS24 | 1.3 | (1-1.6) | 0.016 |
| PCDH15 | 0.93 | (0.87-1) | 0.035 |
| FCRL2 | 0.9 | (0.84-0.97) | 0.0046 |
| RAB11FIP1 | 0.84 | (0.72-0.99) | 0.04 |
| SCUBE1 | 0.91 | (0.83-0.99) | 0.023 |
| CCNL2 | 0.85 | (0.74-0.98) | 0.022 |
| DOCK8 | 0.85 | (0.74-0.98) | 0.022 |
| TLR10 | 0.84 | (0.77-0.92) | 0.00024 |
| FCRL5 | 0.92 | (0.86-0.99) | 0.032 |
| MS4A8B | 0.95 | (0.91-0.99) | 0.026 |
| GSG2 | 1.3 | (1.1-1.4) | 0.00066 |
| PRAM1 | 0.89 | (0.8-0.98) | 0.022 |
| HVCN1 | 0.79 | (0.66-0.94) | 0.0079 |
| EMR3 | 0.88 | (0.82-0.95) | 0.0016 |
| TUBA1C | 1.4 | (1.1-1.7) | 0.0016 |
| COL27A1 | 0.9 | (0.82-0.99) | 0.033 |
| TSLP | 0.9 | (0.83-0.97) | 0.011 |
| WNT3A | 0.92 | (0.86-0.98) | 0.016 |
| FGD3 | 0.78 | (0.67-0.9) | 0.0005 |
| FAM125B | 0.75 | (0.63-0.88) | 0.00035 |
| CEACAM21 | 0.88 | (0.79-0.97) | 0.013 |
| C15orf23 | 1.3 | (1.1-1.5) | 0.0011 |
| SEC11C | 0.87 | (0.77-0.99) | 0.029 |
| HN1L | 1.3 | (1-1.7) | 0.037 |
| IL33 | 0.9 | (0.82-0.99) | 0.027 |
| MUC16 | 1.1 | (1-1.1) | 0.017 |
| SFXN1 | 1.5 | (1.2-1.9) | 0.00027 |
| SIGLEC11 | 0.87 | (0.79-0.95) | 0.0018 |
| C1QTNF7 | 0.88 | (0.81-0.95) | 0.0021 |
| RASGRP4 | 0.88 | (0.79-0.99) | 0.036 |
| CMTM5 | 0.86 | (0.77-0.96) | 0.0079 |
| CLNK | 0.85 | (0.78-0.94) | 0.0011 |
| GYLTL1B | 0.84 | (0.76-0.92) | 0.00033 |
| AMICA1 | 0.83 | (0.74-0.94) | 0.0021 |
| LRIG3 | 0.87 | (0.77-0.98) | 0.02 |
| WFIKKN2 | 0.83 | (0.77-0.91) | 0.000035 |
| DCBLD2 | 1.1 | (1-1.3) | 0.018 |
| COL6A6 | 0.89 | (0.83-0.96) | 0.0016 |
| IL34 | 0.9 | (0.81-1) | 0.04 |
| CD300LF | 0.87 | (0.78-0.96) | 0.0073 |
| CD300LG | 0.94 | (0.88-1) | 0.049 |
| GIMAP8 | 0.84 | (0.72-0.97) | 0.017 |
| CLEC12A | 0.9 | (0.83-0.99) | 0.021 |
| GIMAP7 | 0.84 | (0.72-0.96) | 0.014 |
| SLC5A9 | 0.92 | (0.85-1) | 0.047 |
| SLFNL1 | 0.88 | (0.81-0.96) | 0.0034 |
| RSPH9 | 0.91 | (0.83-0.99) | 0.027 |
| SDK1 | 0.9 | (0.83-0.97) | 0.0087 |
| PCSK9 | 1.1 | (1-1.1) | 0.019 |
| MAMDC2 | 0.93 | (0.87-1) | 0.035 |
| STEAP2 | 1.1 | (1-1.2) | 0.045 |
| IZUMO1 | 0.86 | (0.78-0.96) | 0.0051 |
| IGSF10 | 0.88 | (0.81-0.96) | 0.0034 |
| TREML1 | 0.85 | (0.77-0.94) | 0.0011 |
| COL28A1 | 0.93 | (0.88-0.99) | 0.028 |
| CYP26C1 | 1.1 | (1-1.2) | 0.023 |
| RAET1G | 1.1 | (1-1.2) | 0.034 |
| SLCO4C1 | 0.91 | (0.84-0.98) | 0.01 |
| LRRC33 | 0.83 | (0.72-0.97) | 0.017 |
| GIMAP6 | 0.84 | (0.73-0.98) | 0.023 |
| LOC541473 | 0.89 | (0.81-0.97) | 0.012 |
| SFTPA2 | 0.96 | (0.92-1) | 0.04 |

Figure S1: PPI network inferred by STRING using 436 differentially expressed immune genes with p.value<0.05 in cox test. Hub genes were identified by cytoscape. Shown is cluster1 with 31 genes.


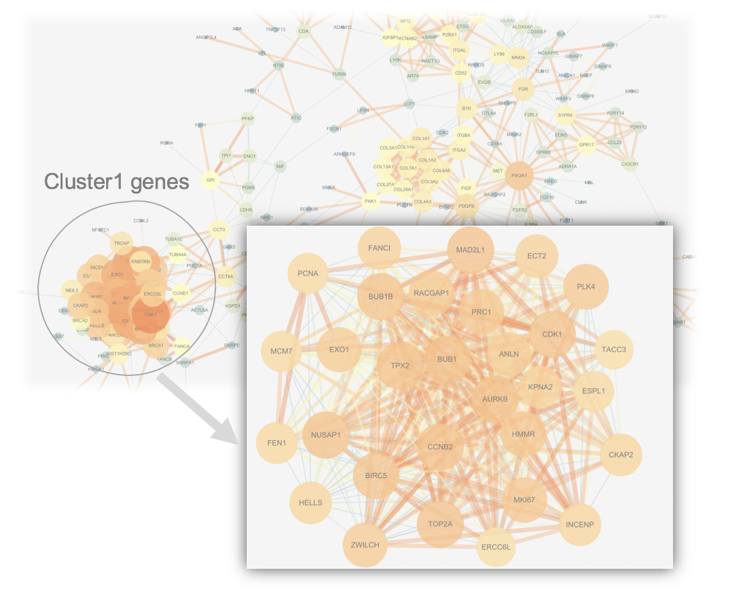


Figure S2. Mutation landscape of 436 target genes (top50) in 569 tumors
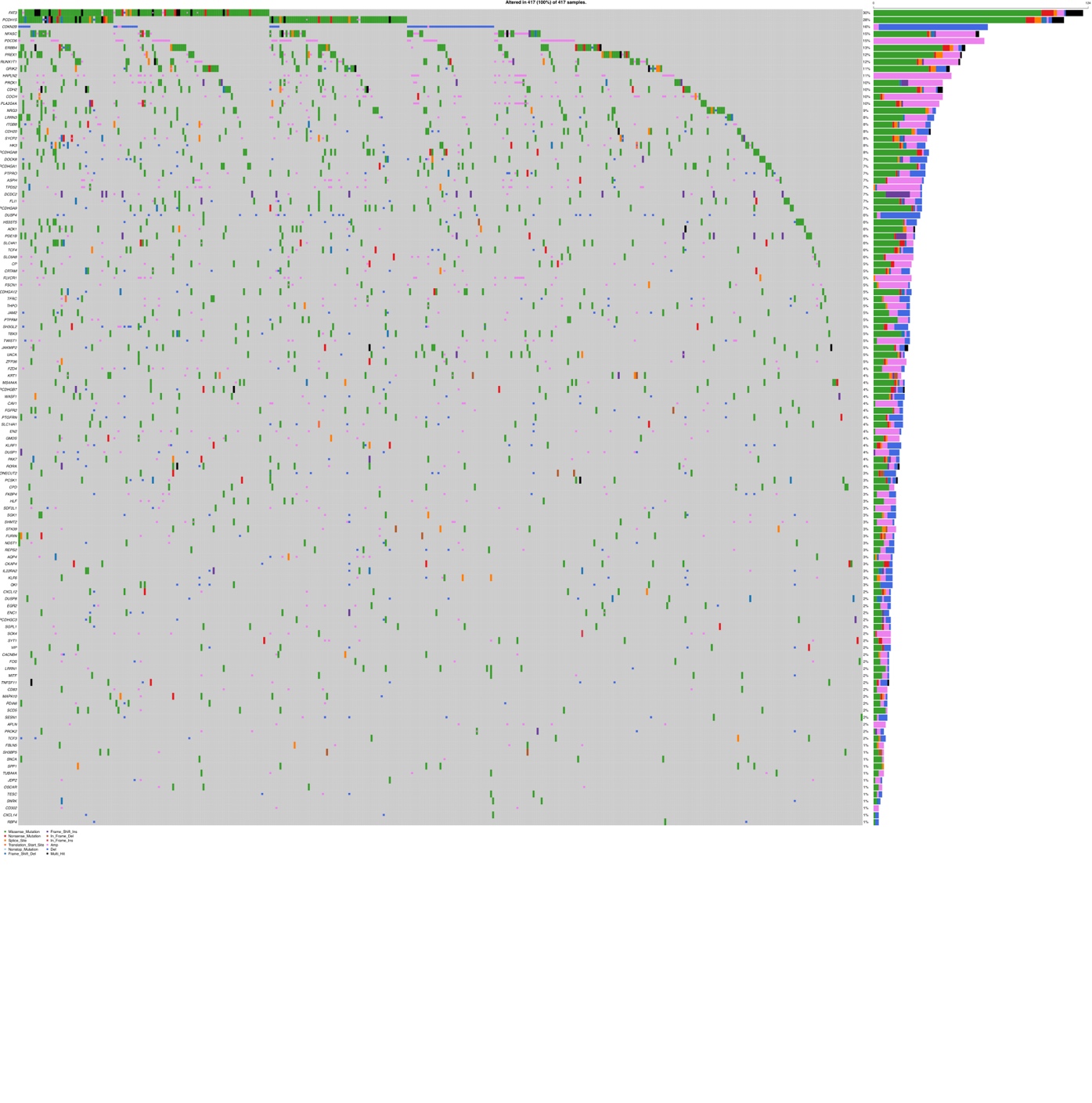


Figure S3. The coefficients for the genes retained in model 4


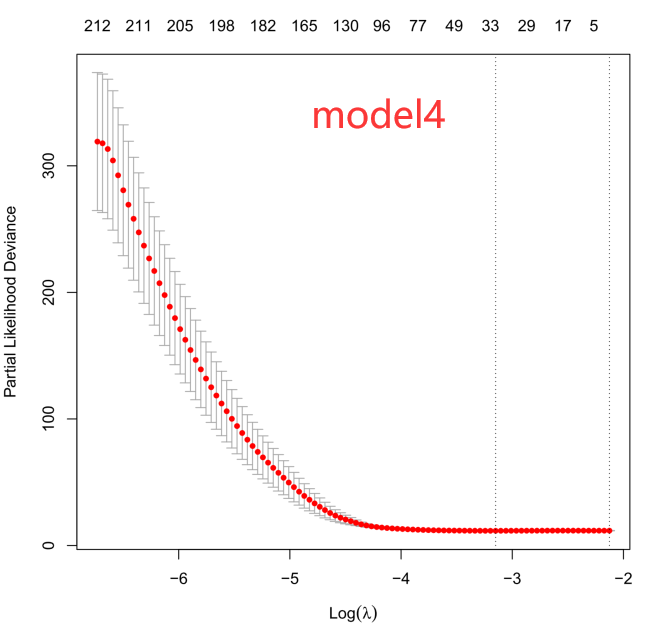


Table S2: The list of coefficients for genes retained in model 4

|  | gene_var | Coef |
| --- | --- | --- |
| Model4 | CAMP | -0.01692 |
|  | CCT6A | 0.12492 |
|  | CDH17 | 0.027753 |
|  | EFNB2 | 0.063466 |
|  | FKBP3 | 0.168033 |
|  | GATA2 | -0.04737 |
|  | ITIH4 | -0.0019 |
|  | SMAD9 | -0.00976 |
|  | P2RX1 | -0.05753 |
|  | PFKP | 0.005527 |
|  | PKP2 | 0.000291 |
|  | PTGFRN | 0.165327 |
|  | PTPRH | 0.012224 |
|  | CCL20 | 0.044296 |
|  | SSR4 | -0.00496 |
|  | KLF10 | 0.020462 |
|  | UPK1B | 0.022307 |
|  | SLC7A5 | 0.015813 |
|  | FKBP6 | -0.03473 |
|  | FERMT2 | 0.242851 |
|  | FLRT1 | 0.126247 |
|  | DDIT4 | 0.099631 |
|  | LY6K | 0.015394 |
|  | NLRP2 | -0.03022 |
|  | HAPLN2 | 0.085175 |
|  | CCNL2 | -0.01797 |
|  | EMR3 | -0.02903 |
|  | COL27A1 | -0.02567 |
|  | TSLP | -0.05821 |
|  | SFXN1 | 0.001779 |
|  | WFIKKN2 | -0.00517 |
|  | PCSK9 | 0.031454 |
|  | IZUMO1 | -0.02983 |

Figure S4. Identification of an immune signature predicting prognosis risk of patients in LUAD using each models（A1,B1,C1: A cutoff of risk factor for modle 1, modle 2, modle 3; A2,B2,C2: Survival analysis of the training dataset for modle 1, modle 2, modle 3; A3,B3,C3: Survival analysis in the testing data for modle 1, modle 2, modle 3.）


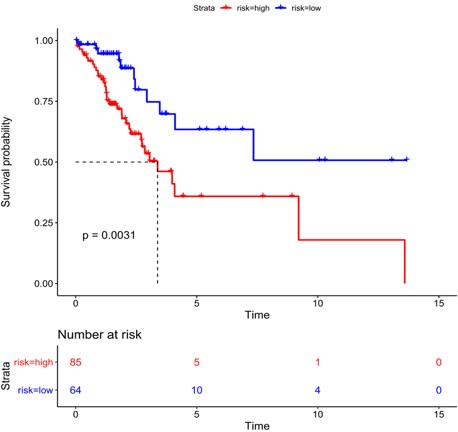

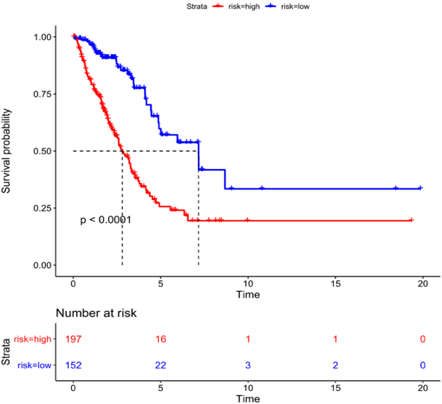

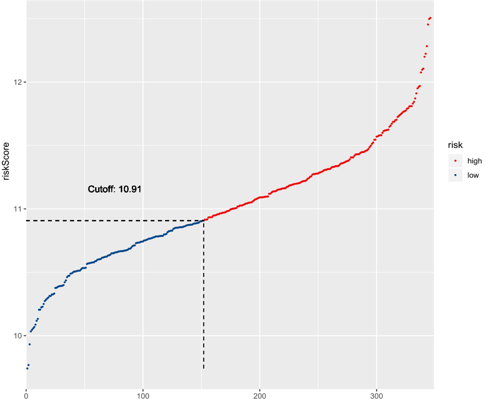


**C1**

**B1**

**A1**


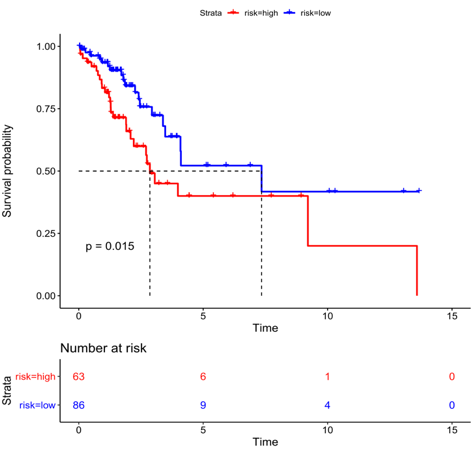

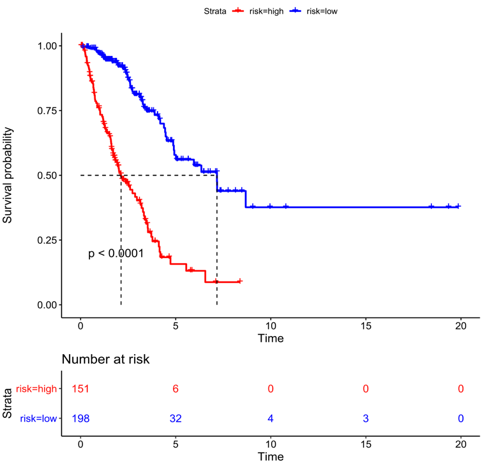

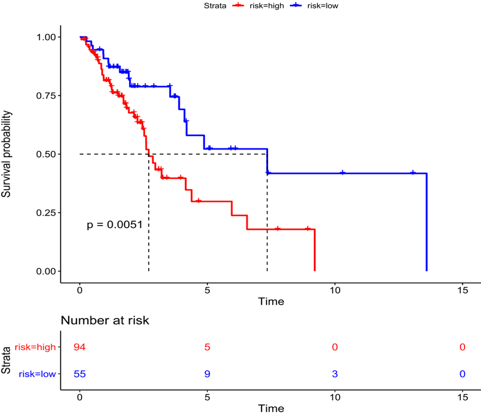

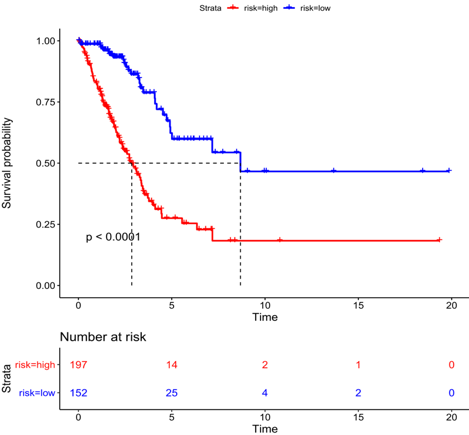

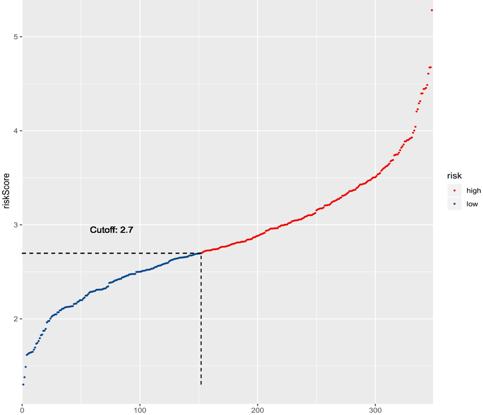


**C2**

**C3**

**A3**

**B3**

**B2**

**A2**


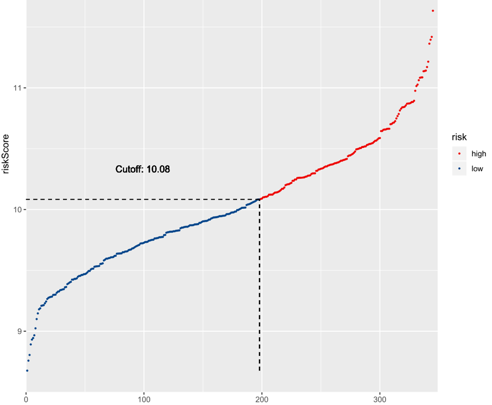


Figure S5. Relationships between the risk score and CD8+ T cell markers

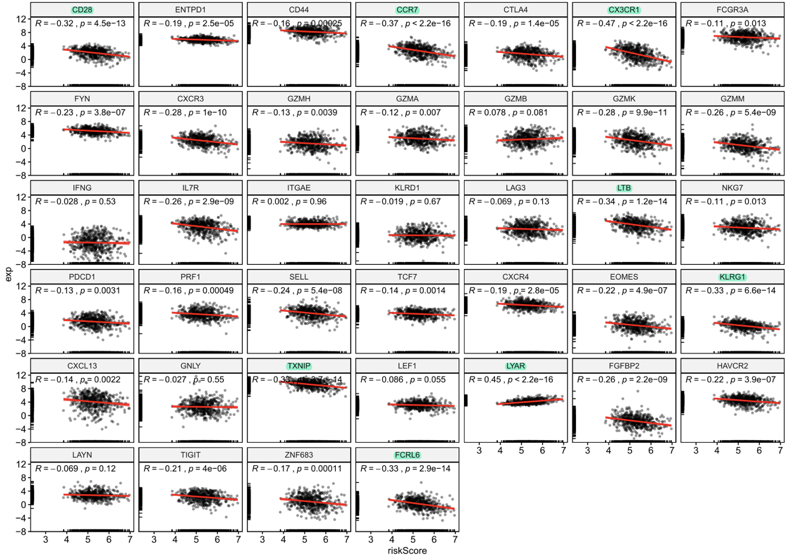


Figure S6. Relationships between the risk score and CD4+ T cell markers


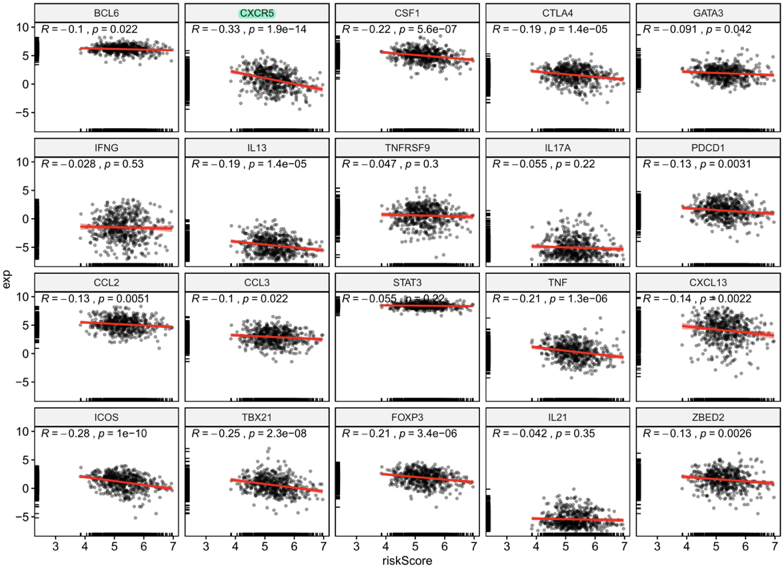


Figure S7. Relationships between the risk score and B cell markers


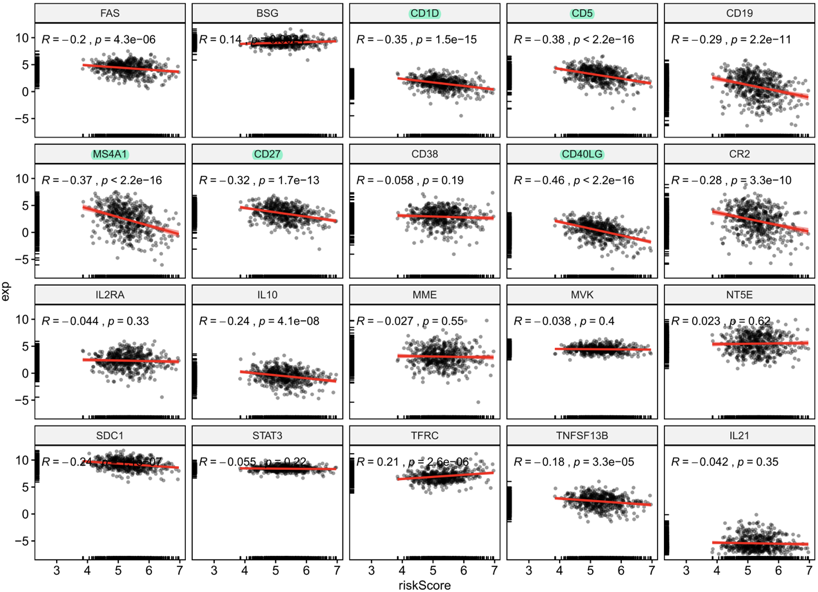


Figure S8. Relationships between the risk score and dendritic cell markers


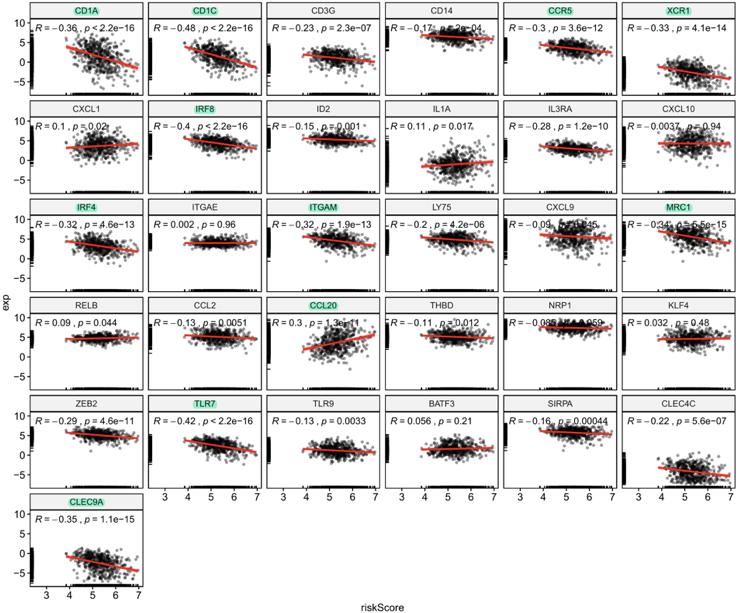

Supplement: Supplementary file 1 — Additional file 1: Table S1. A total of 436 differentially expressed genes were identified. Figure S1. PPI network inferred by STRING using 436 differentially expressed immune genes with p.value < 0.05 in cox test. Hub genes were identified by cytoscape. Shown is cluster1 with 31 genes. Figure S2. Mutation landscape of 436 target genes (top50) in 569 tumors. Figure S3. The coefficients for the genes retained in model 4. Table S2. The list of coefficients for genes retained in model 4. Figure S4. Identification of an immune signature predicting prognosis risk of patients in LUAD using each models (A1,B1,C1: A cutoff of risk factor for modle 1, modle 2, modle 3; A2,B2,C2: Survival analysis of the training dataset for modle 1, modle 2, modle 3; A3,B3,C3: Survival analysis in the testing data for modle 1, modle 2, modle 3). Figure S5. Relationships between the risk score and CD8+ T cell markers. Figure S6. Relationships between the risk score and CD4+ T cell markers. Figure S7. Relationships between the risk score and B cell markers. Figure S8. Relationships between the risk score and dendritic cell markers. [file 12885_2021_7911_MOESM1_ESM.docx]
